# Supplementary material for: Genomic and physiological signatures of adaptation in pathogenic fungi
Source: Nat Commun. 2026 Jan 15;17:748. doi: 10.1038/s41467-026-68330-6 (PMC12820176; doi:10.1038/s41467-026-68330-6)
Supplement: Supplementary file 1 — Supplementary Information [file 41467_2026_68330_MOESM1_ESM.pdf]

Supplementary Information

Genomic and physiological signatures of adaptation in pathogenic fungi

Marco Alexandre Guerreiro[1,2,*], Andrey Yurkov[3], Minou Nowrousian[4], Kirk Broders[5], Eva H. Stukenbrock[1,2]

[1] Environmental Genomics Group, Botanical Institute, Christian-Albrechts University of Kiel, Kiel, Germany

[2] Max Planck Institute for Evolutionary Biology, Plön, Germany

[3] Leibniz Institute DSMZ-German Collection of Microorganisms and Cell Cultures, Braunschweig, Germany

[4] Department of Molecular and Cellular Botany, Ruhr University Bochum, Bochum, Germany

[5] USDA, Agricultural Research Service, National Center for Agricultural Utilization Research, Mycotoxin Prevention and Applied Microbiology Research Unit, 1815 N. University, Peoria, IL. 61604, U.S.A

*** Corresponding author**: Marco Alexandre Guerreiro, Max Planck Institute for Evolutionary Biology, August-Thienemann-Str. 2, 24306 Plön and Christian-Albrechts University of Kiel, Am Botanischen Garten 1-9, 24118 Kiel

Phone: +49 (0) 431 880 6366, Fax: +49 (0) 431 880 6369

Email: mguerreiro@bot.uni-kiel.de

Supplementary Discussion

Convergent tRNA gene expansion and evolution

As reported for other eukaryotes, including fungi and bacteria [1–3], genomes of *Trichosporonales* species only use a subset of the full theoretical set of 61 tRNA gene families (Supplementary Fig. 6 and Supplementary Fig. 7). The total number of distinct tRNA gene families (i.e., anticodon types) reported in previous studies correspond to the numbers that we predict for *Trichosporonales* (between 41 and 46). This is above the theoretical minimum of 30 anticodons for decoding the standard genetic code [1]. Interestingly, some codons and amino acids detected in protein-coding genes in our study were missing complementary tRNAs in the genome (Supplementary Fig. 7 and Supplementary Data 8). Previous studies have reported that codon usage and codon abundance in protein-coding genes do not always correspond to the most abundant associated tRNA gene, and some tRNAs may even remain undetected in the genome [2,4]. It has been suggested that in fungi, tRNA wobbling of the third base position might enable a tRNA to recognize several unspecific synonymous codons and thereby allow the decoding of these amino acids [2,4,5]. The absence of some tRNA could also be due to technical limitations of predictions tools, such as classification of tRNAs as pseudogenised genes (Supplementary Data 7) or incorrect assignment of the isotype.

Studies report that some non-coding RNAs have regulatory roles independent of their primary functions (e.g. tRNA, mRNA) [6–8]. Variable tRNA gene sequences are hypothesised to confer unique roles but currently these remain undefined [9]. Similar to reports in other eukaryotes, including fungi [9], we identified high intragenomic sequence variation for tRNA genes having the same anticodon sequence (Supplementary Fig. 11, Supplementary Fig. 12 and Supplementary Fig. 13). This variation was predominant in low-copy tRNA gene families. Evolutionary experiments in *Saccharomyces cerevisiae* revealed that the tRNA pool within the genome can rapidly evolve to meet translational demands [10]. Furthermore, mutations in tRNA genes are a common adaptive mechanism to obtain

higher fitness when presented with new environmental challenges [10]. Our findings highlight that the evolution of individual tRNA gene families is characterized by different selective pressures.

The increase in the tRNA gene copy number is thought to be the result of genome expansion [11], which is supported by our results (Supplementary Fig. 15). In some species, tRNA expansion has been linked directly to lifestyle transitions such as in the *Sordariomycetes*, where tRNA expansion has been associated with pathogenic lifestyles [12]. tRNA expansions also are a response to changes in codon usage [13], which, as found here, could imply adaptation of species to new ecological niches, namely association with hosts.

Mechanisms driving tRNA gene copy number and codon composition are currently poorly understood [14]. Analyses combining phylogenetic information and tRNA numbers indicate that the expansion of these genes is possibly a result of convergent evolution (Supplementary Fig. 8 and Supplementary Fig. 9). Correlative and similarity analyses based on the structure of the tRNA pool (i.e., relative abundance of each gene family) support convergent evolutionary expansion of some tRNA genes along the phylogeny. Convergent evolution is generally attributed to shared molecular, genetic, physiological, or ecological constraints, which can limit or bias the genomic or phenotypic variation among organisms [16]. Alternatively, convergent evolution can be a result of stochastic processes, albeit to a lesser extent [16]. The convergence of codon usage frequencies and optimization at a macroevolutionary level may be an indication of shared constraints, which are imposed by both neutral and adaptive pressures [3]. Further research is needed to clarify the mechanisms driving tRNA gene copy number and codon composition during evolutionary processes. This knowledge may provide insights into the adaptive mechanisms that influence tRNA expansion and convergence, while contributing to our understanding of how organisms adapt to new ecological niches.

Supplementary Figures

Supplementary Figure 1. Assessment of proteome completeness. Asterisks (*) indicate genome assemblies that, due to high content of duplicated BUSCOs, are suspected to be hybrids or diploid genomes and were therefore excluded from further analyses. BUSCO analyses were performed on predicted proteomes.

Supplementary Figure 2. Ancestral state reconstruction of lifestyle. The pie charts at nodes indicate the likelihood of each possible ancestral state for lifestyle transitions. Lifestyles are color-coded for saprotrophic (blue) and opportunistic pathogenic (orange) states.

Supplementary Figure 3. Correlations between genome size, repeat content (**A**), and transposable element (TE) content (**B**). Linear regressions (red line) and phylogenetically independent contrast (PICs) correlations (Spearman's Rho and *P* value) are indicated.

Supplementary Figure 4. Comparison of genomic features between opportunistic pathogenic and saprotrophic lifestyles within *Apiotrichum*, *Cryptococcus*, *Cutaneotrichosporon*, and *Trichosporon* genera. The genome size (A), GC content (B), number of predicted tRNA genes (C), repeat (D) and TE content (E) in the genome, the total number of predicted proteins (F), secreted proteins (G), CAZymes (H) secreted CAZymes (I), carbohydrate-related genes (J) and lipid-related genes (K) was compared between pathogenic and saprotrophic species within each genus. No significant statistical differences ($P \geq 0.05$) were observed based on the Wilcoxon signed-rank test due to the low number of samples. To avoid overestimation, only 1 genome per species was considered. The total number of considered strains is indicated. Each boxplot indicates the median value (center line), the 25th and 75th percentiles (lower and upper hinges), and the most extreme data points (whiskers) within 1.5 × inter-quartile range. Individual points beyond the whiskers are outliers.

Supplementary Figure 5. Distribution of the total number of tRNA genes across 41 fungal genomes of the *Trichosporonales* order.

Supplementary Figure 6. Distribution of unique tRNA anticodon types among *Trichosporonales* species. The dotted grey line represents the median value.

Supplementary Figure 7. Distribution of tRNA gene copy number by anticodon and respective amino acid. Circle shapes are scaled and color-coded for gene copy number. Genera names are abbreviated (*Ap*., *Apiotrichum*; *Pa*., *Pascua*; *Pr*., *Prillingera*; *Cu*., *Cutaneotrichosporon*; *Tr*., *Trichosporon*; *Va*., *Vanrija*; *Ha*., *Haglerozyma*; *Ta*., *Takashimella*; *Cr*., *Cryptococcus*).

Supplementary Figure 8. Variation in the phylogenetic signal (Blomberg's K) of tRNA gene copy number in *Trichosporonales*. A total of 43/45 tRNA genes comprised Blomberg's K not equal to 1 (±0.02), suggesting that they expanded less than (blue) or greater than (red) expected by genetic drift according to the Brownian Motion model of evolution. All signals were statistically significant (P <0.05).

Supplementary Figure 9. tRNA composition per taxonomic level in the *Trichosporonales* order. The pairwise correlation coefficient of individual gene copy number and Bray-Curtis similarity in relation to the respective phylogenetic distance is indicated for members of the same genus (purple) or different genera (green). Each data point represents 1 pairwise comparison among genomes regarding phylogenetic distance and tRNA gene composition. Due to their basal phylogenetic position, *Takashimella* species were excluded from this analysis.

Supplementary Figure 10. Codon usage and anticodon frequency in *Cryptococcus*. The relative synonymous codon usage (RSCU) in the genome is depicted for each amino acid. The relative frequency of the respective decoding tRNA gene family is shown. The results are provided for saprotrophic (*Cryptococcus amylolentus*, *Cryptococcus floricola*) and opportunistic pathogenic

(*Cryptococcus deneoformans* and *Cryptococcus gattii*) species. Codons are color-coded for each amino acid and are independent between amino acids.

Supplementary Figure 11. Intragenomic sequence polymorphism of tRNA genes in relation to copy number (**A**) and by categorical groups (**B**). The genetic distance was calculated for each tRNA gene detected in a single genome. Each data point corresponds to the sequence variability among all copies for the same tRNA gene present in each genome.

Supplementary Figure 12. Intragenomic sequence variation of tRNA genes by anticodon type and respective amino acid.

Supplementary Figure 13. Mean genetic distance among tRNA genes by anticodon and respective amino acid across all *Trichosporonales* species. The plot is sorted by median value. Each boxplot indicates the median value (center line), the 25th and 75th percentiles (lower and upper hinges), and the most extreme data points (whiskers) within 1.5 × inter-quartile range. Individual points beyond the whiskers are outliers.

Supplementary Figure 14. Mean genetic distance among tRNA genes by genome assembly. The plot is sorted by median value. Each boxplot indicates the median value (center line), the 25th and 75th percentiles (lower and upper hinges), and the most extreme data points (whiskers) within 1.5 × inter-quartile range. Individual points beyond the whiskers are outliers.

Supplementary Figure 15. Correlations between the number of tRNA genes and genome size (**A**) or transposable element (TE) content (**B**). Linear regressions and phylogenetically independent contrast (PICs) correlations (Spearman's Rho and *P* value) are indicated for all *Trichosporonales* (black) and lifestyle (orange, opportunistic pathogens; blue, saprotrophic).

Supplementary Figure 16. Comparison of the normalized S on different cellular processes and functions for *Cryptococcus*, *Cutaneotrichosporon*, and *Trichosporon* genera. The S of each function was normalized for each individual against the S of the corresponding genome. Each boxplot indicates the

median value (center line), the 25th and 75th percentiles (lower and upper hinges), and the most extreme data points (whiskers) within 1.5 × inter-quartile range. Individual points beyond the whiskers are outliers.

Supplementary Figure 17. Comparison of codon optimization (S) metrics of saprotrophic (left panel) and opportunistic pathogenic (right panel) species. The distribution of the S values is provided across gene sets (**A**) and on species level (**B**) for 3 different conditions: the absolute S value for carbohydrate or lipid transport and metabolism pathways; the overall S value for the genome; and the normalization of the S value for each pathway in relation to the genomic S value. Each boxplot indicates the median value (center line), the 25th and 75th percentiles (lower and upper hinges), and the most extreme data points (whiskers) within 1.5 × inter-quartile range. Individual points beyond the whiskers are outliers.

Supplementary Figure 18. Relative translation efficiency for genes involved in lipid or carbohydrate transport and metabolism with 1 isolate per species. The translation efficiency (S index) is compared between genera comprising both opportunistic pathogenic and saprotrophic species, except for *Trichosporon*. The opportunistic pathogens were grouped into common (*Cutaneotrichosporon*, *Trichosporon*, and *Cryptococcus*) or emerging (*Apiotrichum*) pathogens. These results consider only 1 genome per species and are consistent with the ones presented and are provided on Figure 3. Each boxplot indicates the median value (center line), the 25th and 75th percentiles (lower and upper hinges), and the most extreme data points (whiskers) within 1.5 × inter-quartile range. Individual points beyond the whiskers are outliers.

Supplementary Figure 19. Direct comparison of the codon optimization (S values) among single-copy orthogroups. Protein sequences of genes involved in carbohydrate and lipid transport and metabolism were clustered into orthogroups. The S value was determined based on single-copy orthogroups present in all tested species with well-defined lifestyles (*Cryptococcus*, *Cutaneotrichosporon*, and *Trichosporon*). The S ratio represents the relative codon optimization between both pathways (S lipid : S carbohydrate) for each species. Each boxplot indicates the median value (center line), the 25th and

75th percentiles (lower and upper hinges), and the most extreme data points (whiskers) within 1.5 × inter-quartile range. Individual points beyond the whiskers are outliers.

Supplementary Figure 20. Relative translation efficiency for genes involved in lipid and carbohydrate transport and metabolism. The relative translation efficiency (S index) is compared between genera and opportunistic pathogenic (OP) and saprotrophic (S) lifestyles. Each boxplot indicates the median value (center line), the 25th and 75th percentiles (lower and upper hinges), and the most extreme data points (whiskers) within 1.5 × inter-quartile range. Individual points beyond the whiskers are outliers.

Supplementary Figure 21. Growth of isolates cultivated at 18°C, 23°C, 28°C, 33°C and 37°C for 10 days. The optical density (OD) values at 600 nm are the mean from 4 replicates and presented excluding the baseline OD of the growth medium. The isolates are separated according to genus and color-coded according to lifestyle. Error bars represent the observed variation among replicates.

Supplementary Figure 22. Growth of isolates cultivated at 18°C, 23°C, 28°C, 33°C and 37°C for 6 days in 5 mL PD medium. Each tube corresponds to a different isolate and temperature. Qualitative growth was evaluated based on turbidity. A Wickerham card (black stripes) was placed behind each tube as a visual aid. Representative examples of growth intensity (no growth, weak, moderate and strong growth) are provided.

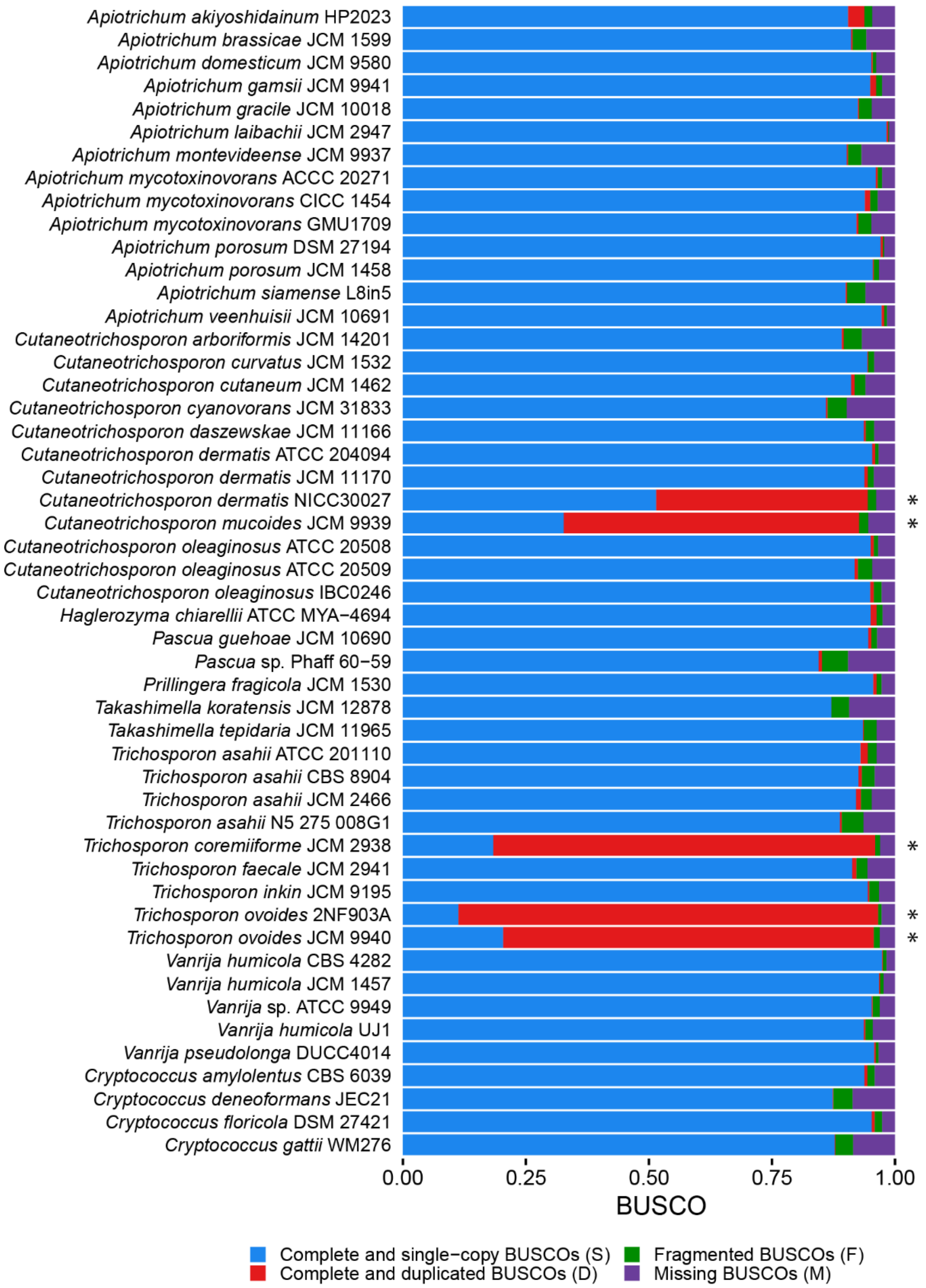

Supplementary Figure 1. Assessment of proteome completeness. Asterisks (*) indicate genome assemblies that, due to high content of duplicated BUSCOs, are suspected to be hybrids or diploid genomes and were therefore excluded from further analyses. BUSCO analyses were performed on predicted proteomes.

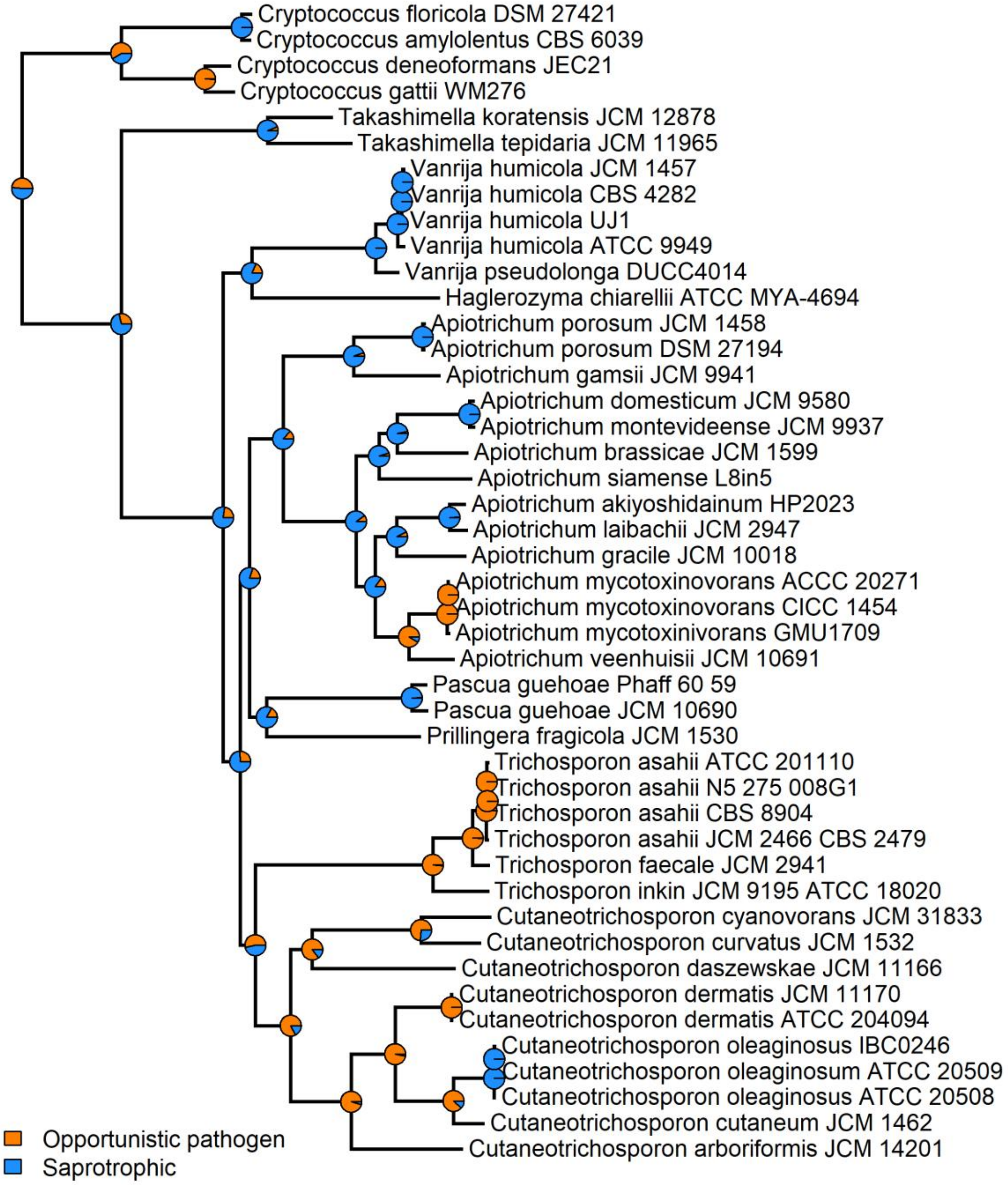

Supplementary Figure 2. Ancestral state reconstruction of lifestyle. The pie charts at nodes indicate the likelihood of each possible ancestral state for lifestyle transitions. Lifestyles are color-coded for saprotrophic (blue) and opportunistic pathogenic (orange) states.

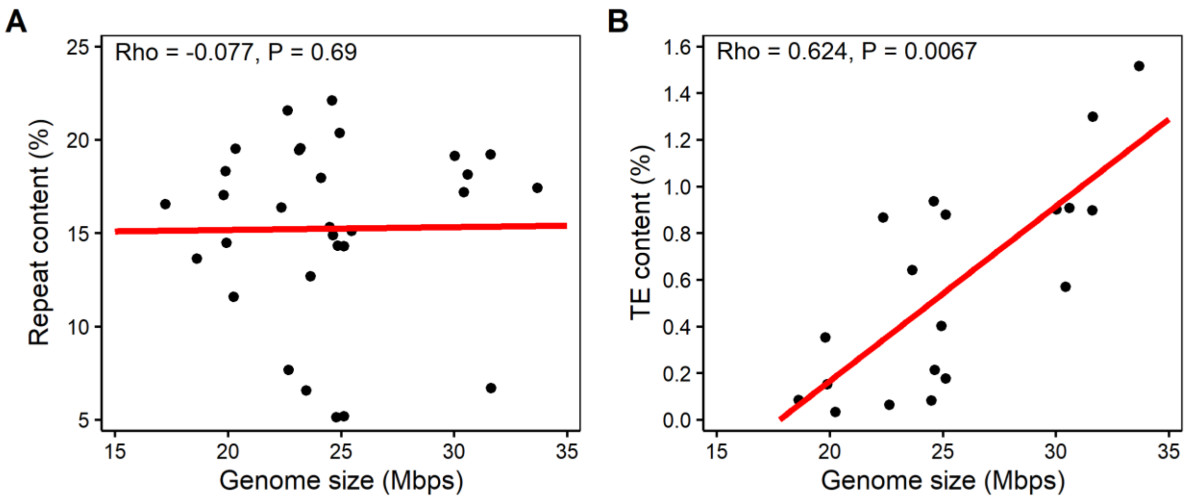

Supplementary Figure 3. Correlations between genome size, repeat content (**A**), and transposable element (TE) content (**B**). Linear regressions (red line) and phylogenetically independent contrast (PICs) correlations (Spearman's Rho and *P* value) are indicated.

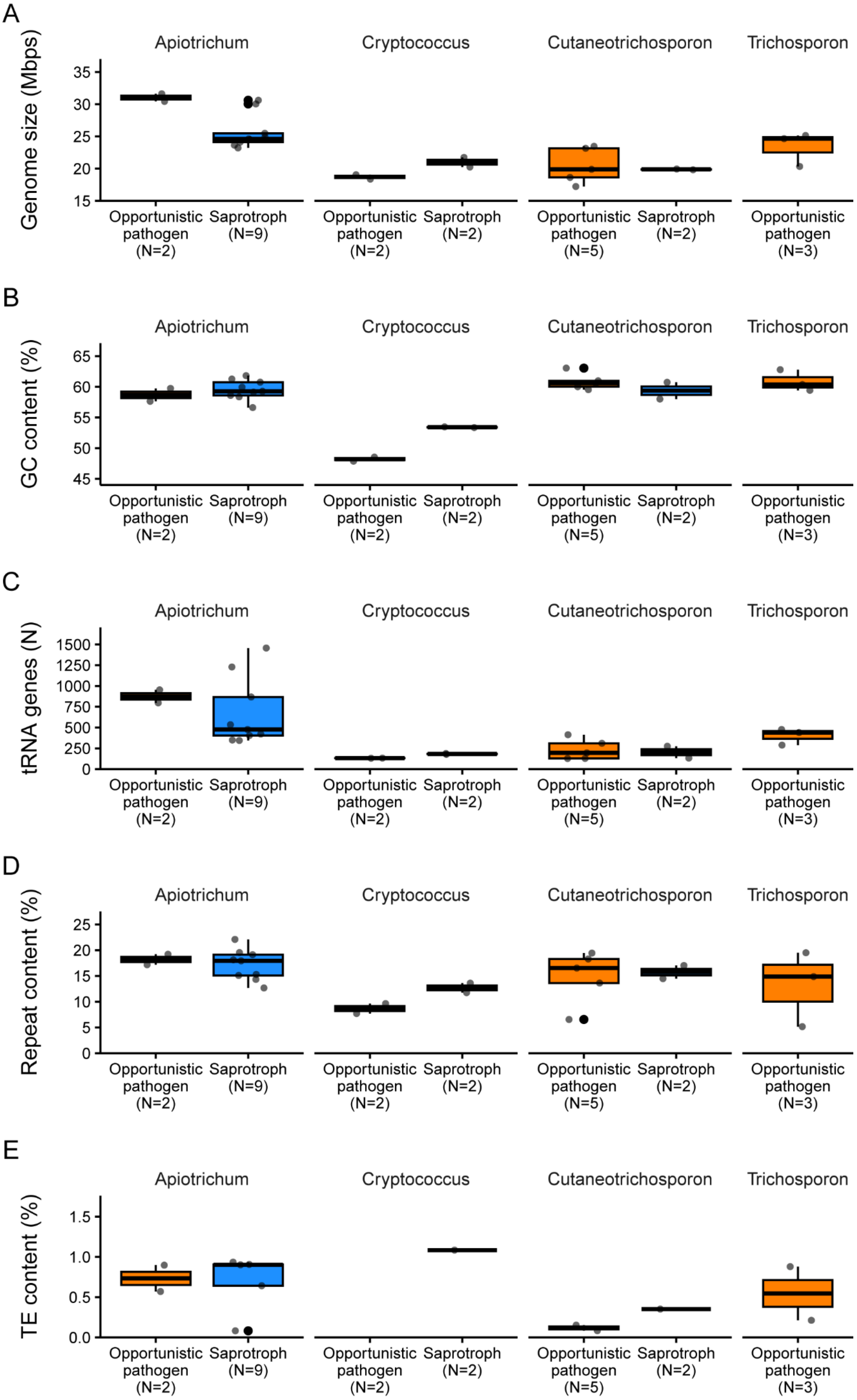
A
B
C
D
E
Apiotrichum
Cryptococcus
Cutaneotrichosporon
Trichosporon
Genome size (Mbps)
GC content (%)
tRNA genes (N)
Repeat content (%)
TE content (%)
Opportunistic pathogen (N=2)
Saprotroph (N=9)
Opportunistic pathogen (N=2)
Saprotroph (N=2)
Opportunistic pathogen (N=5)
Saprotroph (N=2)
Opportunistic pathogen (N=3)

196

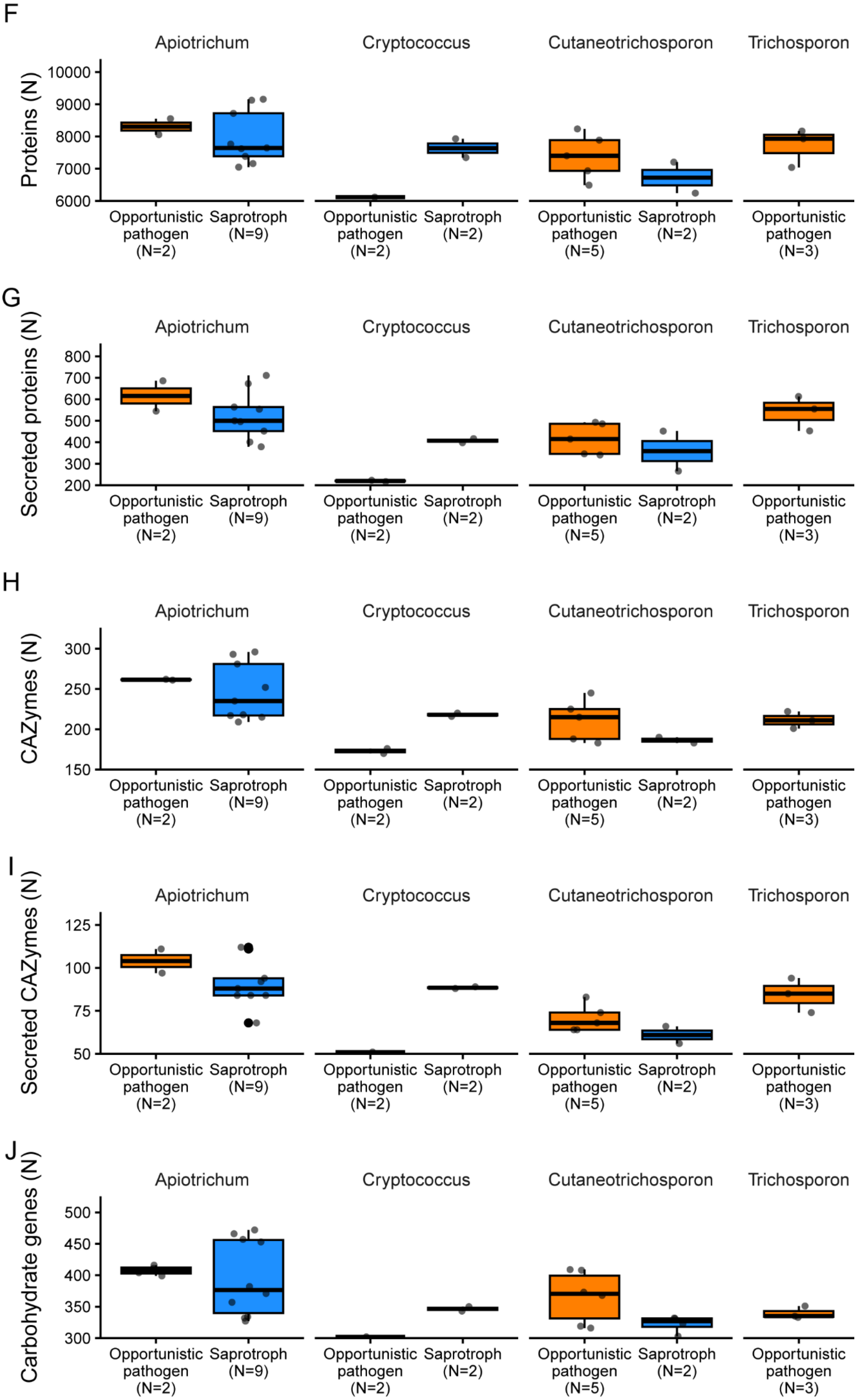
F
G
H
I
J
Apiotrichum
Cryptococcus
Cutaneotrichosporon
Trichosporon
Proteins (N)
Secreted proteins (N)
CAZymes (N)
Secreted CAZymes (N)
Carbohydrate genes (N)
Opportunistic pathogen (N=2)
Saprotroph (N=9)
Opportunistic pathogen (N=2)
Saprotroph (N=2)
Opportunistic pathogen (N=5)
Saprotroph (N=2)
Opportunistic pathogen (N=3)

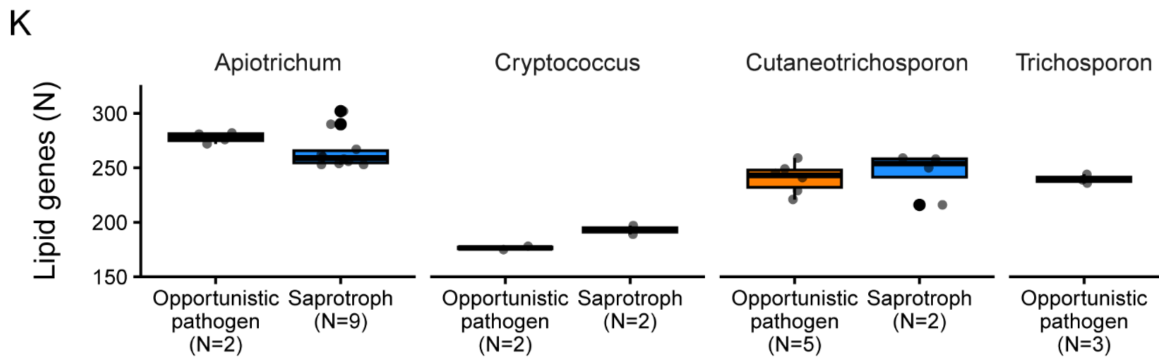

Supplementary Figure 4. Comparison of genomic features between opportunistic pathogenic and saprotrophic lifestyles within *Apiotrichum*, *Cryptococcus*, *Cutaneotrichosporon*, and *Trichosporon* genera. The genome size (A), GC content (B), number of predicted tRNA genes (C), repeat (D) and TE content (E) in the genome, the total number of predicted proteins (F), secreted proteins (G), CAZymes (H) secreted CAZymes (I), carbohydrate-related genes (J) and lipid-related genes (K) was compared between pathogenic and saprotrophic species within each genus. No significant statistical differences ($P \geq 0.05$) were observed based on the Wilcoxon signed-rank test due to the low number of samples. To avoid overestimation, only 1 genome per species was considered. The total number of considered strains is indicated. Each boxplot indicates the median value (center line), the 25th and 75th percentiles (lower and upper hinges), and the most extreme data points (whiskers) within 1.5 × inter-quartile range. Individual points beyond the whiskers are outliers.

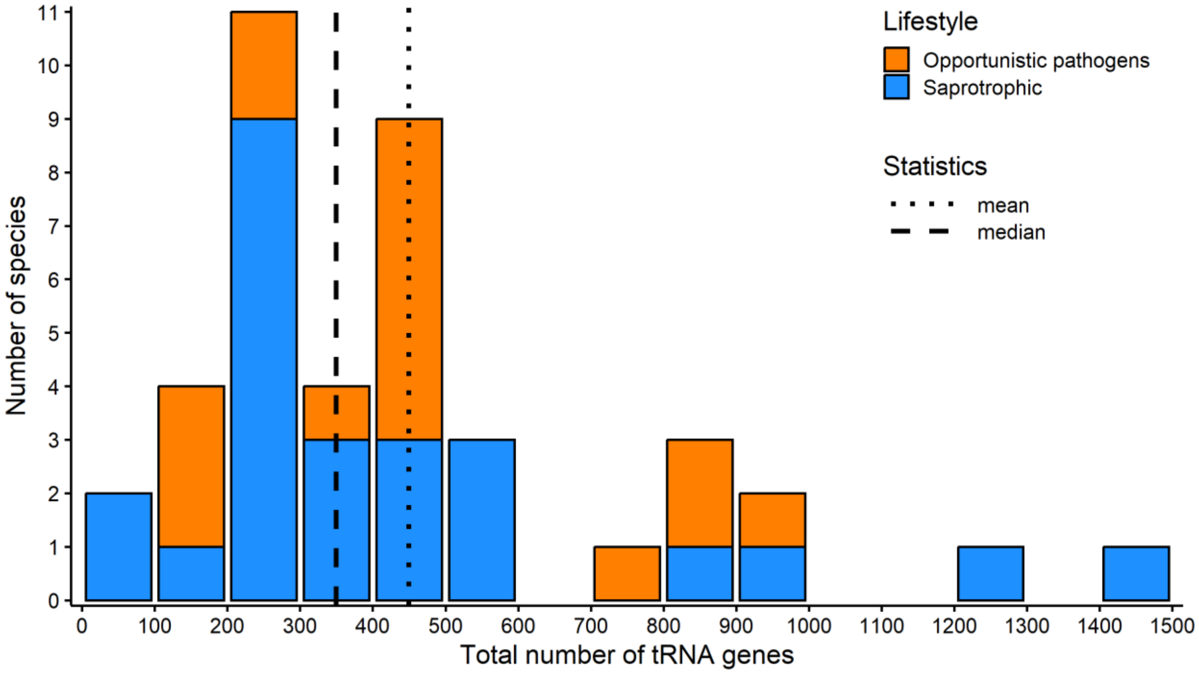

Supplementary Figure 5. Distribution of the total number of tRNA genes across 41 fungal genomes of the *Trichosporonales* order.

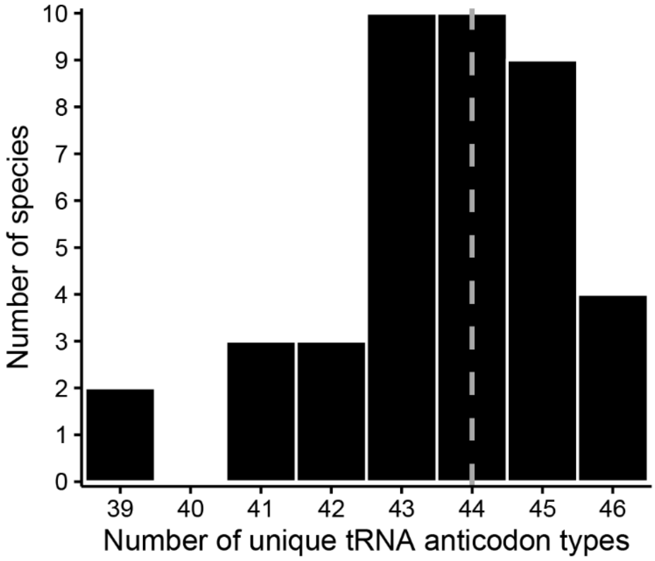

Supplementary Figure 6. Distribution of unique tRNA anticodon types among *Trichosporonales* species. The dotted grey line represents the median value.

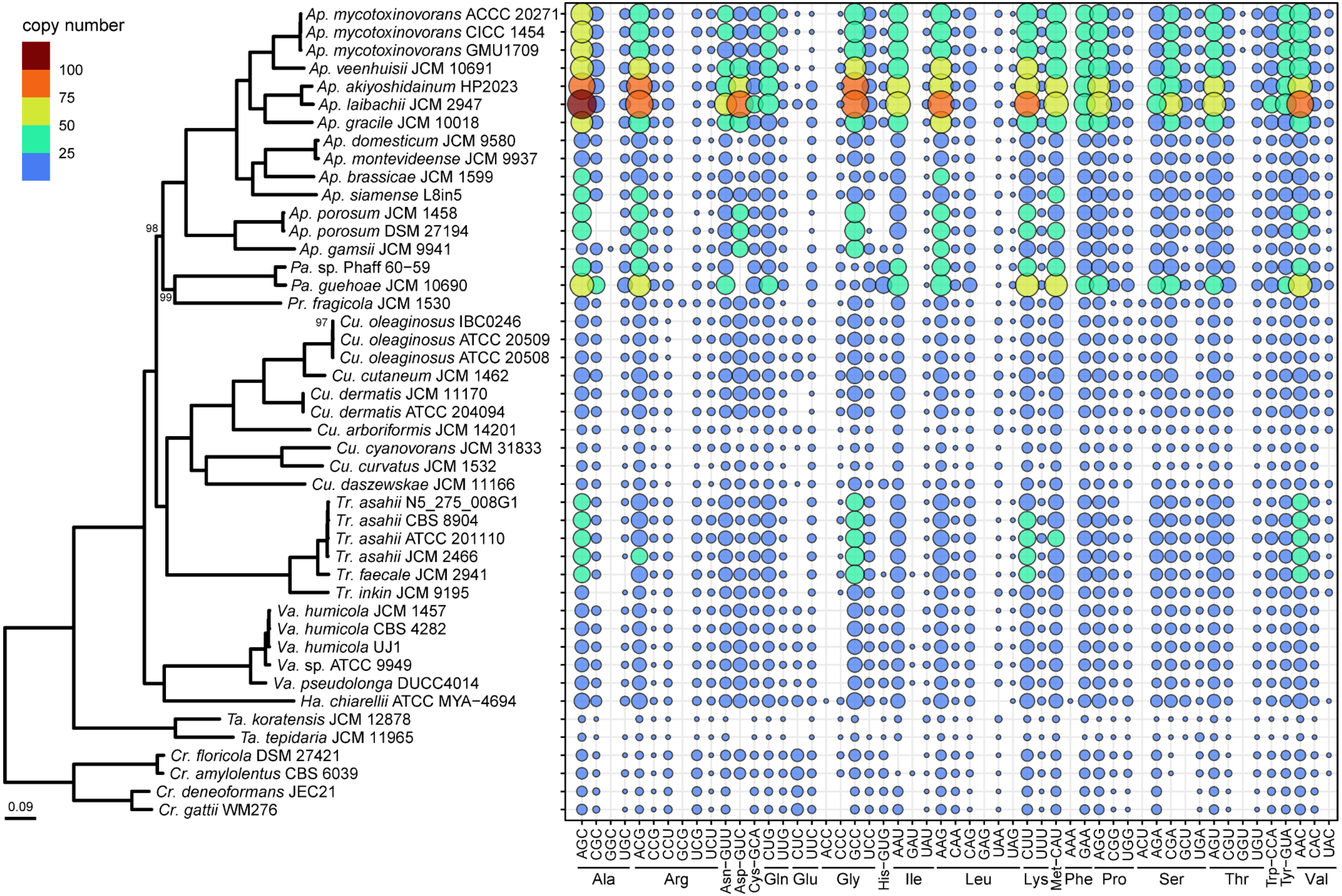
copy number
100
75
50
25
98
99
97
0.09
Ap. mycotoxinovorans ACCC 20271
Ap. mycotoxinovorans CICC 1454
Ap. mycotoxinovorans GMU1709
Ap. veenhuisii JCM 10691
Ap. akiyoshidainum HP2023
Ap. laibachii JCM 2947
Ap. gracile JCM 10018
Ap. domesticum JCM 9580
Ap. montevideense JCM 9937
Ap. brassicae JCM 1599
Ap. siamense L8in5
Ap. porosum JCM 1458
Ap. porosum DSM 27194
Ap. gamsii JCM 9941
Pa. sp. Phaff 60−59
Pa. guehoae JCM 10690
Pr. fragicola JCM 1530
Cu. oleaginosus IBC0246
Cu. oleaginosus ATCC 20509
Cu. oleaginosus ATCC 20508
Cu. cutaneum JCM 1462
Cu. dermatis JCM 11170
Cu. dermatis ATCC 204094
Cu. arboriformis JCM 14201
Cu. cyanovorans JCM 31833
Cu. curvatus JCM 1532
Cu. daszewskae JCM 11166
Tr. asahii N5_275_008G1
Tr. asahii CBS 8904
Tr. asahii ATCC 201110
Tr. asahii JCM 2466
Tr. faecale JCM 2941
Tr. inkin JCM 9195
Va. humicola JCM 1457
Va. humicola CBS 4282
Va. humicola UJ1
Va. sp. ATCC 9949
Va. pseudolonga DUCC4014
Ha. chiarellii ATCC MYA−4694
Ta. koratensis JCM 12878
Ta. tepidaria JCM 11965
Cr. floricola DSM 27421
Cr. amylolentus CBS 6039
Cr. deneoformans JEC21
Cr. gattii WM276
AGC CGC GGC UGC ACG CCG CCU GCG UCG UCU Asn−GUU Asp−GUC Cys−GCA CUG UUG CUC UUC ACC GCC UCC His−GUG AAU GAU UAU AAG CAA CAG GAG UAA UAG CUU UUU Met−CAU AAA GAA AGG CGG UGG ACU AGA CGA GCU UGA AGU CGU GGU UGU Trp−CCA Tyr−GUA AAC CAC UAC
Ala
Arg
Gln
Glu
Gly
Ile
Leu
Lys
Phe
Pro
Ser
Thr
Val

Supplementary Figure 7. Distribution of tRNA gene copy number by anticodon and respective amino acid. Circle shapes are scaled and color-coded for gene copy number. Genera names are abbreviated (*Ap*., *Apiotrichum*; *Pa*., *Pascua*; *Pr*., *Prillingera*; *Cu*., *Cutaneotrichosporon*; *Tr*., *Trichosporon*; *Va*., *Vanrija*; *Ha*., *Haglerozyma*; *Ta*., *Takashimella*; *Cr*., *Cryptococcus*).

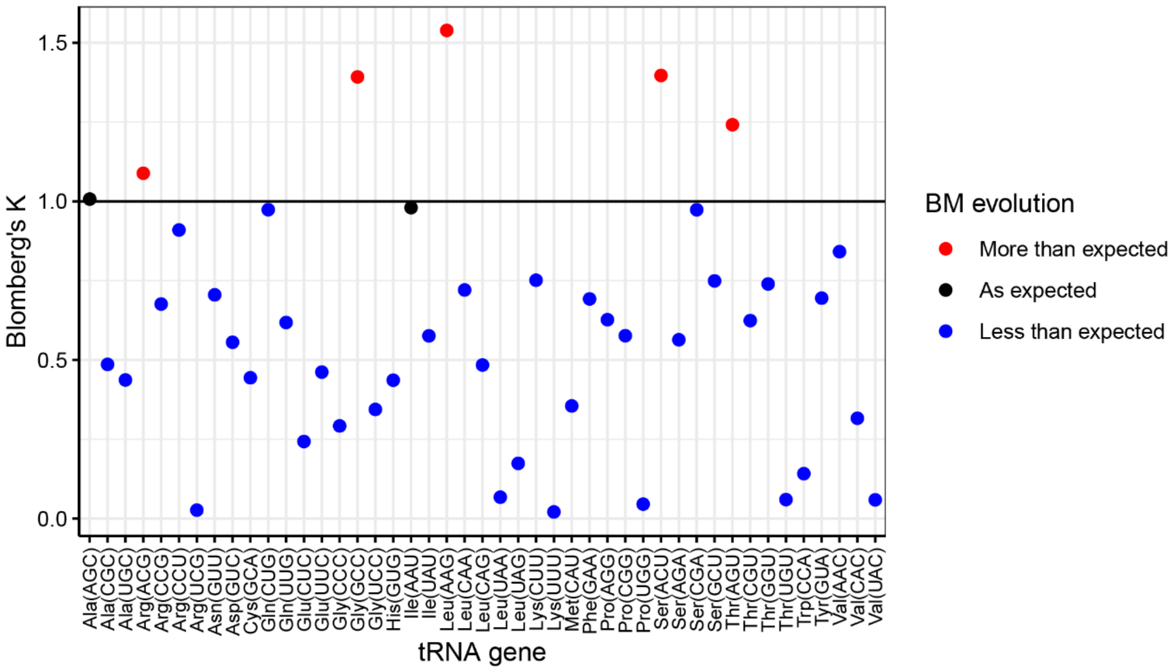

Supplementary Figure 8. Variation in the phylogenetic signal (Blomberg's K) of tRNA gene copy number in *Trichosporonales*. A total of 43/45 tRNA genes comprised Blomberg's K not equal to 1 (±0.02), suggesting they expanded less than (blue) or greater than (red) expected by genetic drift according to the Brownian Motion model of evolution. All phylogenetic signals represented are statistically significant (P <0.05).

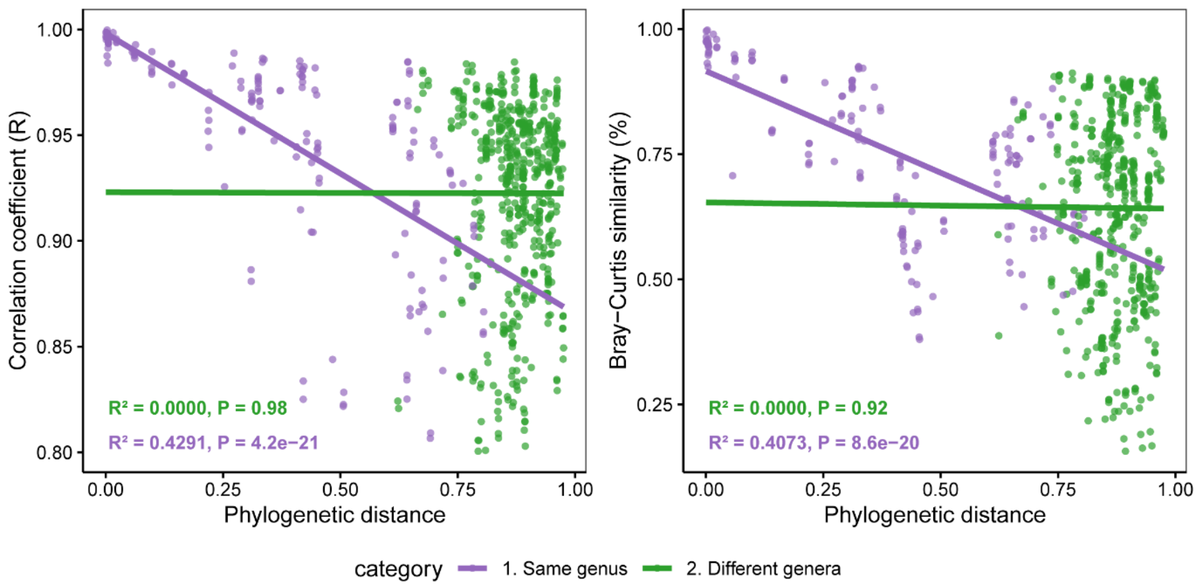

Supplementary Figure 9. tRNA composition per taxonomic level in the *Trichosporonales* order. The pairwise correlation coefficient of individual gene copy number and Bray-Curtis similarity in relation to the respective phylogenetic distance is indicated for members of the same genus (purple) or different genera (green). Each data point represents 1 pairwise comparison among genomes regarding phylogenetic distance and tRNA gene composition. Due to their basal phylogenetic position, *Takashimella* species were excluded from this analysis.

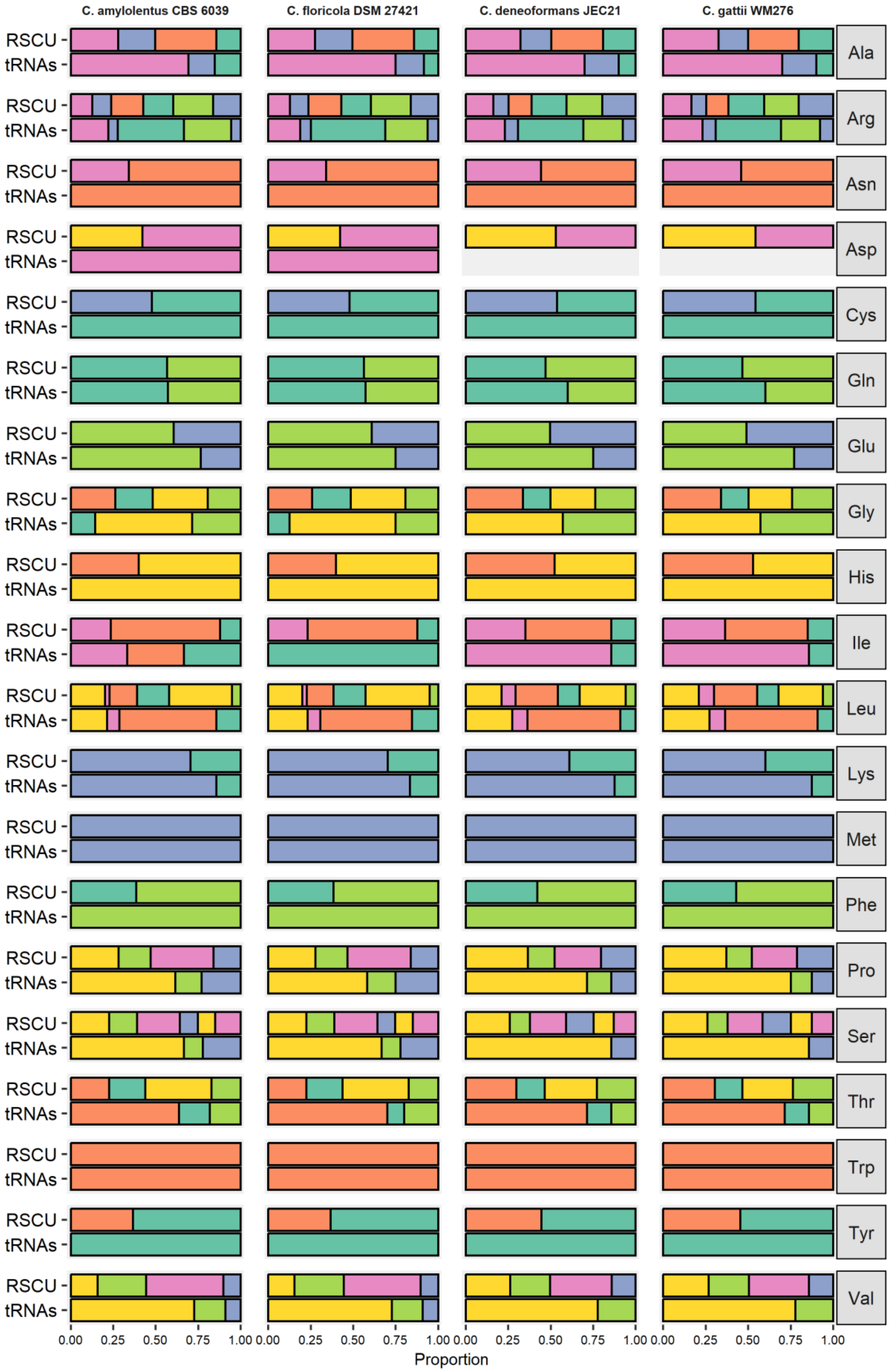
C. amylolentus CBS 6039
C. floricola DSM 27421
C. deneoformans JEC21
C. gattii WM276
RSCU
tRNAs
Ala
Arg
Asn
Asp
Cys
Gln
Glu
Gly
His
Ile
Leu
Lys
Met
Phe
Pro
Ser
Thr
Trp
Tyr
Val
0.00 0.25 0.50 0.75 1.00
Proportion

234

Supplementary Figure 10. Codon usage and anticodon frequency in *Cryptococcus*. The relative synonymous codon usage (RSCU) in the genome is depicted for each amino acid. The relative frequency of the respective decoding tRNA gene family is shown. The results are provided for saprotrophic (*Cryptococcus amylolentus*, *Cryptococcus floricola*) and opportunistic pathogenic (*Cryptococcus deneoformans* and *Cryptococcus gattii*) species. Codons are color-coded for each amino acid and are independent between amino acids.

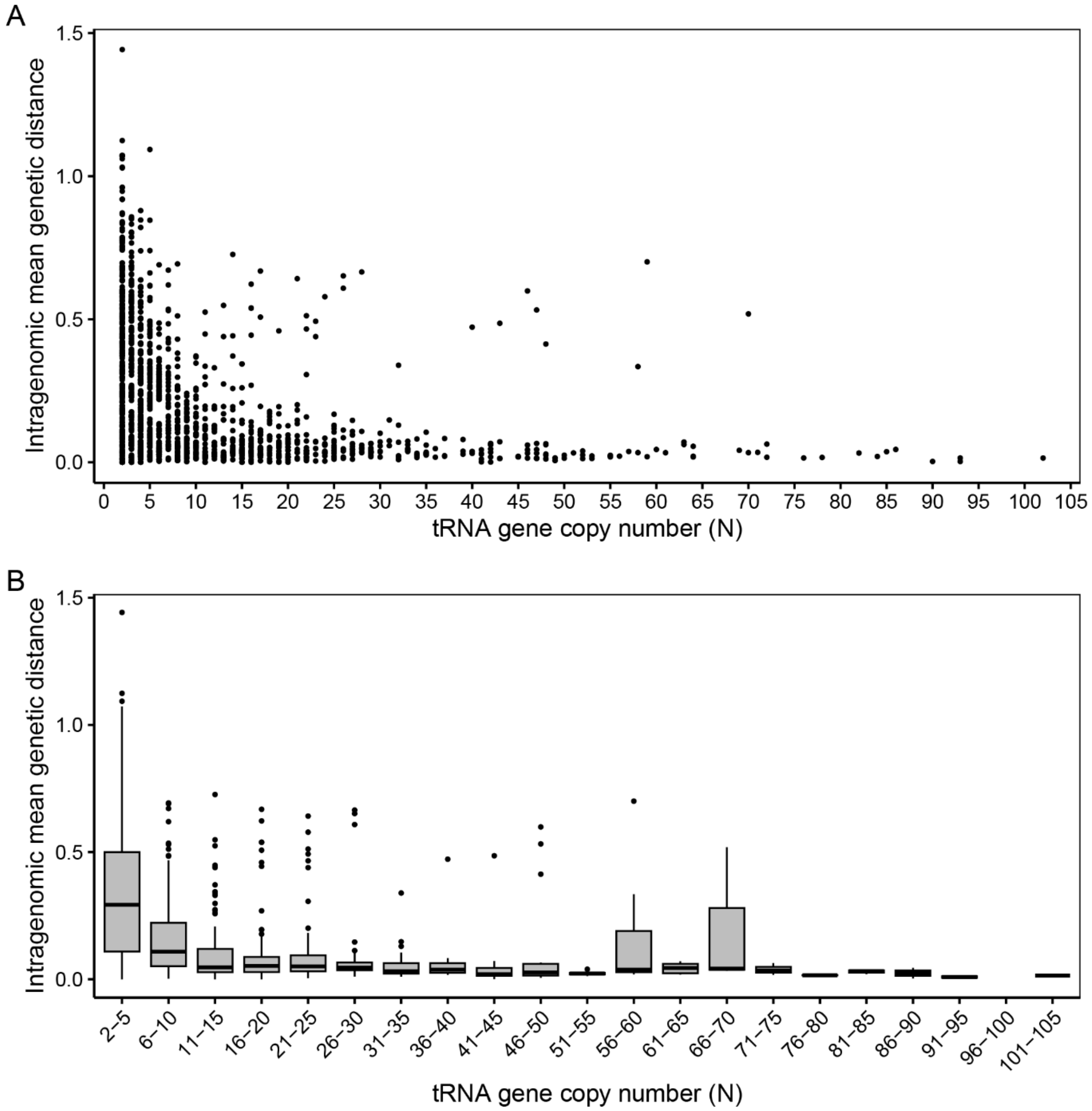

Supplementary Figure 11. Intragenomic sequence polymorphism of tRNA genes in relation to copy number (**A**) and by categorical groups (**B**). The genetic distance was calculated for each tRNA gene detected in a single genome. Each data point corresponds to the sequence variability among all copies for the same tRNA gene present in each genome.

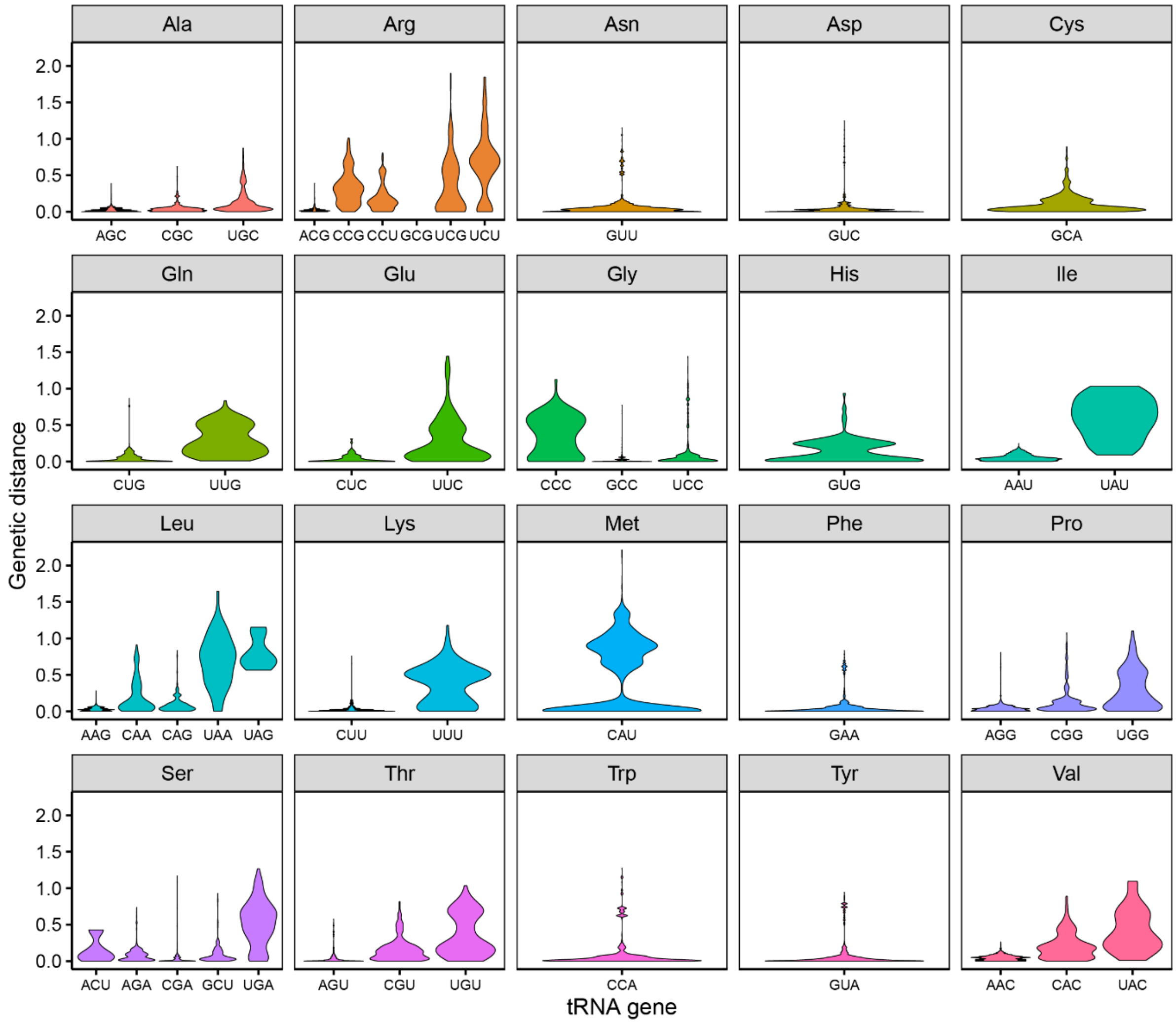

Supplementary Figure 12. Intragenomic sequence variation of tRNA genes by anticodon type and respective amino acid.

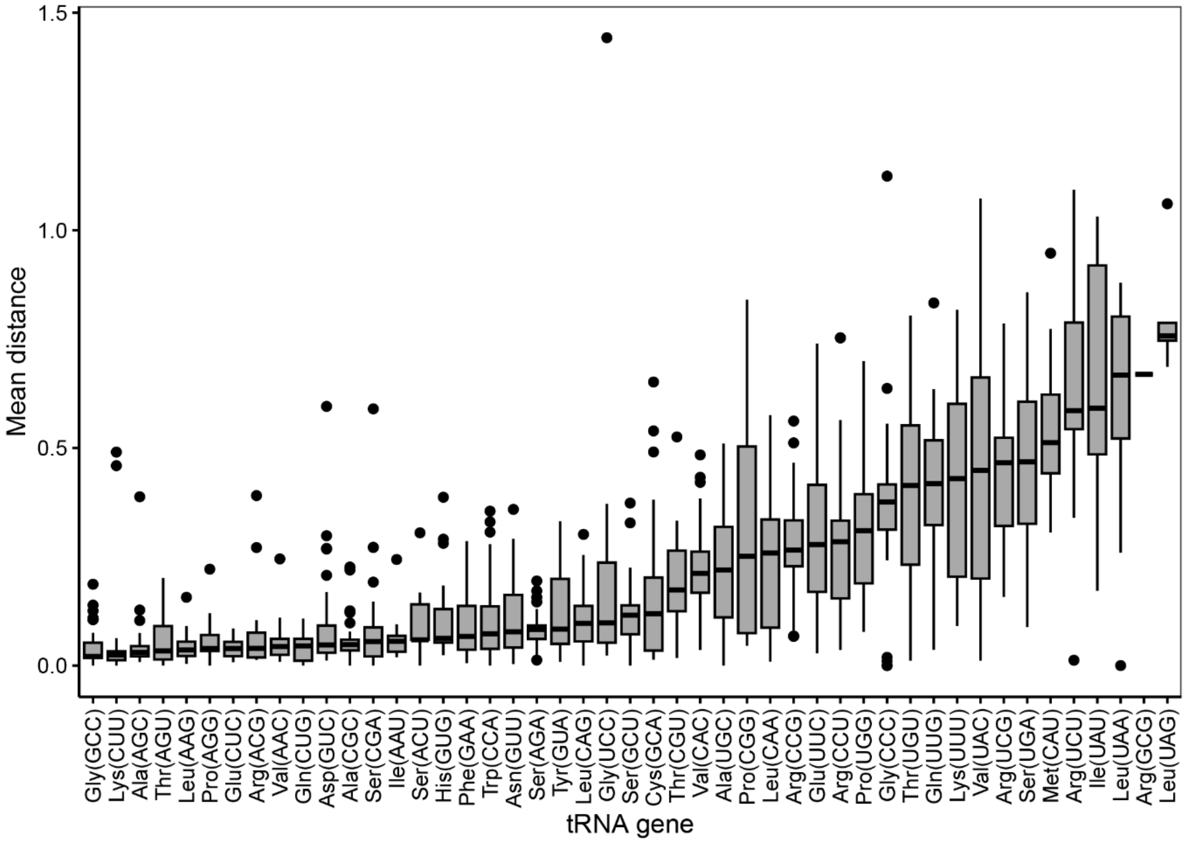

Supplementary Figure 13. Mean genetic distance among tRNA genes by anticodon and respective amino acid across all *Trichosporonales* species. The plot is sorted by median value. Each boxplot indicates the median value (center line), the 25th and 75th percentiles (lower and upper hinges), and the most extreme data points (whiskers) within 1.5 × inter-quartile range. Individual points beyond the whiskers are outliers.

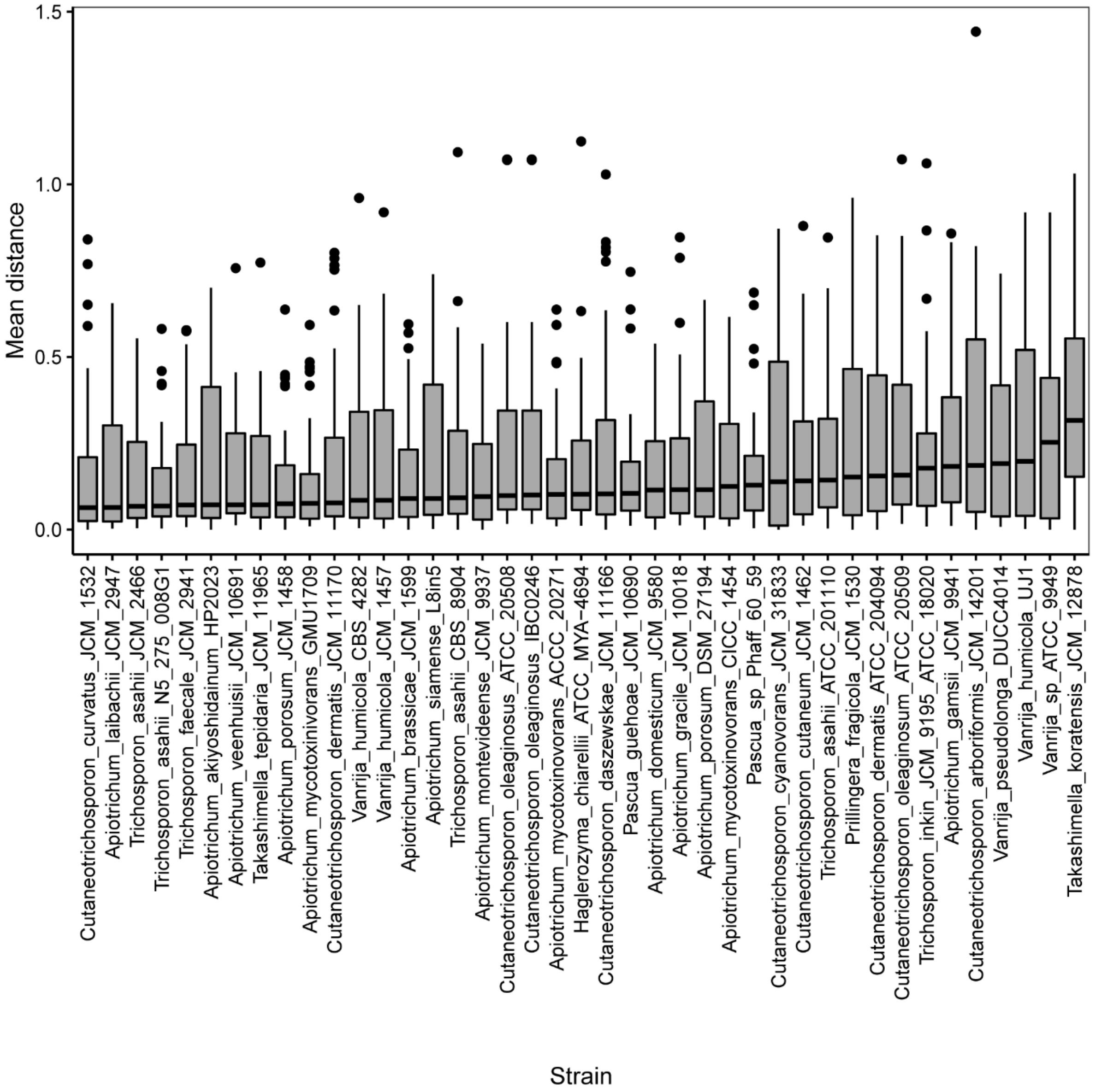

Supplementary Figure 14. Mean genetic distance among tRNA genes by genome assembly. The plot is sorted by median value. Each boxplot indicates the median value (center line), the 25th and 75th percentiles (lower and upper hinges), and the most extreme data points (whiskers) within 1.5 × inter-quartile range. Individual points beyond the whiskers are outliers.

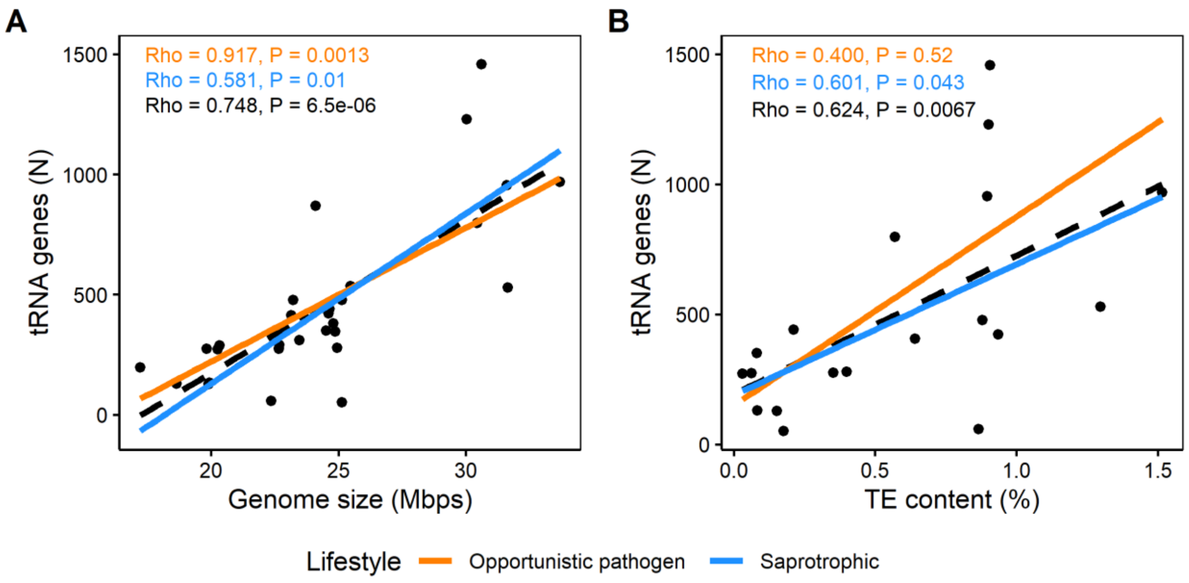

Supplementary Figure 15. Correlations between the number of tRNA genes and genome size (**A**) or transposable element (TE) content (**B**). Linear regressions and phylogenetically independent contrast (PICs) correlations (Spearman's Rho and *P* value) are indicated for all *Trichosporonales* (black) and lifestyle (orange, opportunistic pathogens; blue, saprotrophic).

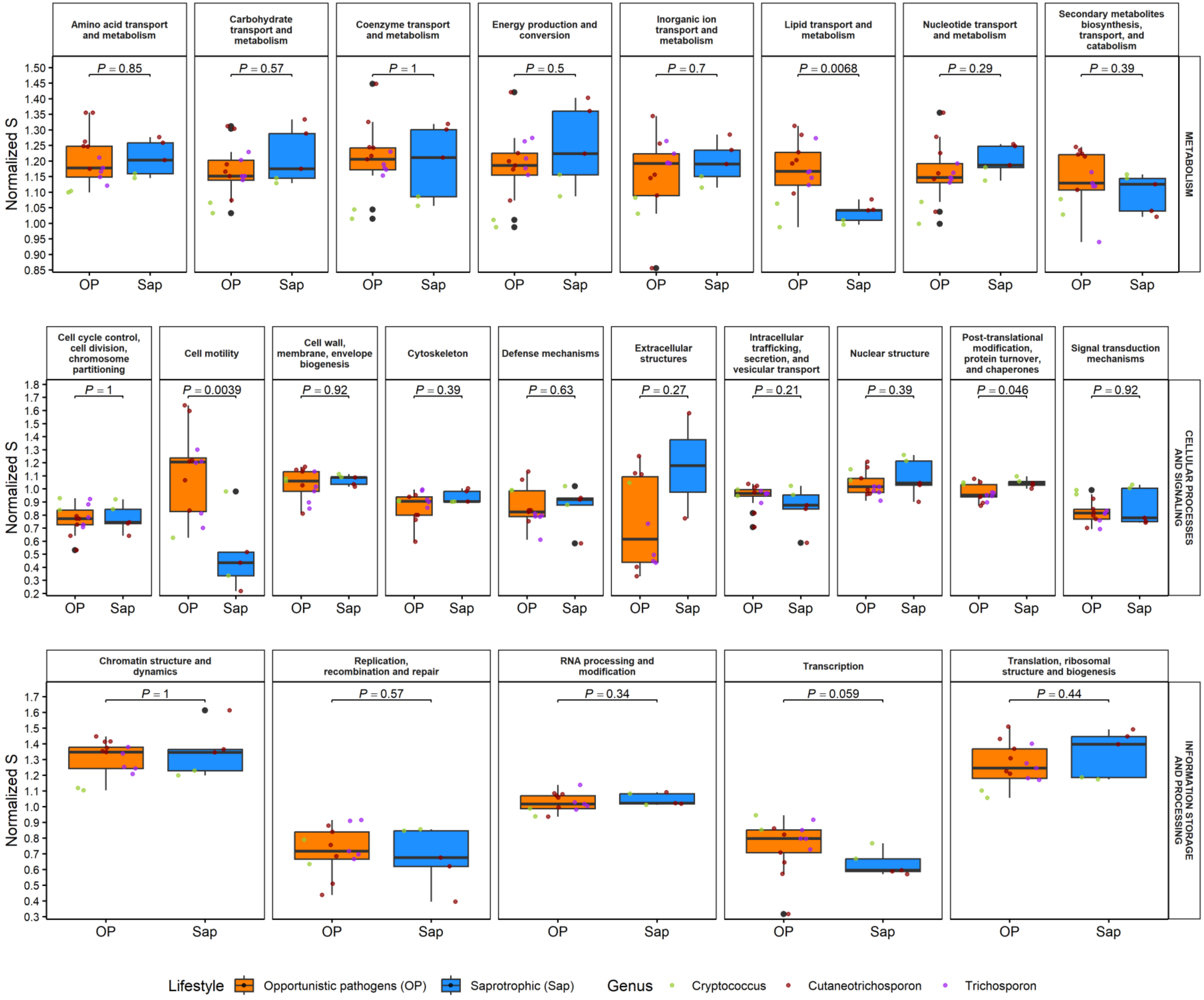
Amino acid transport and metabolism
Carbohydrate transport and metabolism
Coenzyme transport and metabolism
Energy production and conversion
Inorganic ion transport and metabolism
Lipid transport and metabolism
Nucleotide transport and metabolism
Secondary metabolites biosynthesis, transport, and catabolism
P = 0.85
P = 0.57
P = 1
P = 0.5
P = 0.7
P = 0.0068
P = 0.29
P = 0.39
METABOLISM
Cell cycle control, cell division, chromosome partitioning
Cell motility
Cell wall, membrane, envelope biogenesis
Cytoskeleton
Defense mechanisms
Extracellular structures
Intracellular trafficking, secretion, and vesicular transport
Nuclear structure
Post-translational modification, protein turnover, and chaperones
Signal transduction mechanisms
P = 1
P = 0.0039
P = 0.92
P = 0.39
P = 0.63
P = 0.27
P = 0.21
P = 0.39
P = 0.046
P = 0.92
CELLULAR PROCESSES AND SIGNALING
Chromatin structure and dynamics
Replication, recombination and repair
RNA processing and modification
Transcription
Translation, ribosomal structure and biogenesis
P = 1
P = 0.57
P = 0.34
P = 0.059
P = 0.44
INFORMATION STORAGE AND PROCESSING
Normalized S
OP
Sap
Lifestyle
Opportunistic pathogens (OP)
Saprotrophic (Sap)
Genus
Cryptococcus
Cutaneotrichosporon
Trichosporon

265

Supplementary Figure 16. Comparison of the normalized S on different cellular processes and functions for *Cryptococcus*, *Cutaneotrichosporon*, and *Trichosporon* genera. The S of each function was normalized for each individual against the S of the corresponding genome. Each boxplot indicates the median value (center line), the 25th and 75th percentiles (lower and upper hinges), and the most extreme data points (whiskers) within 1.5 × inter-quartile range. Individual points beyond the whiskers are outliers.

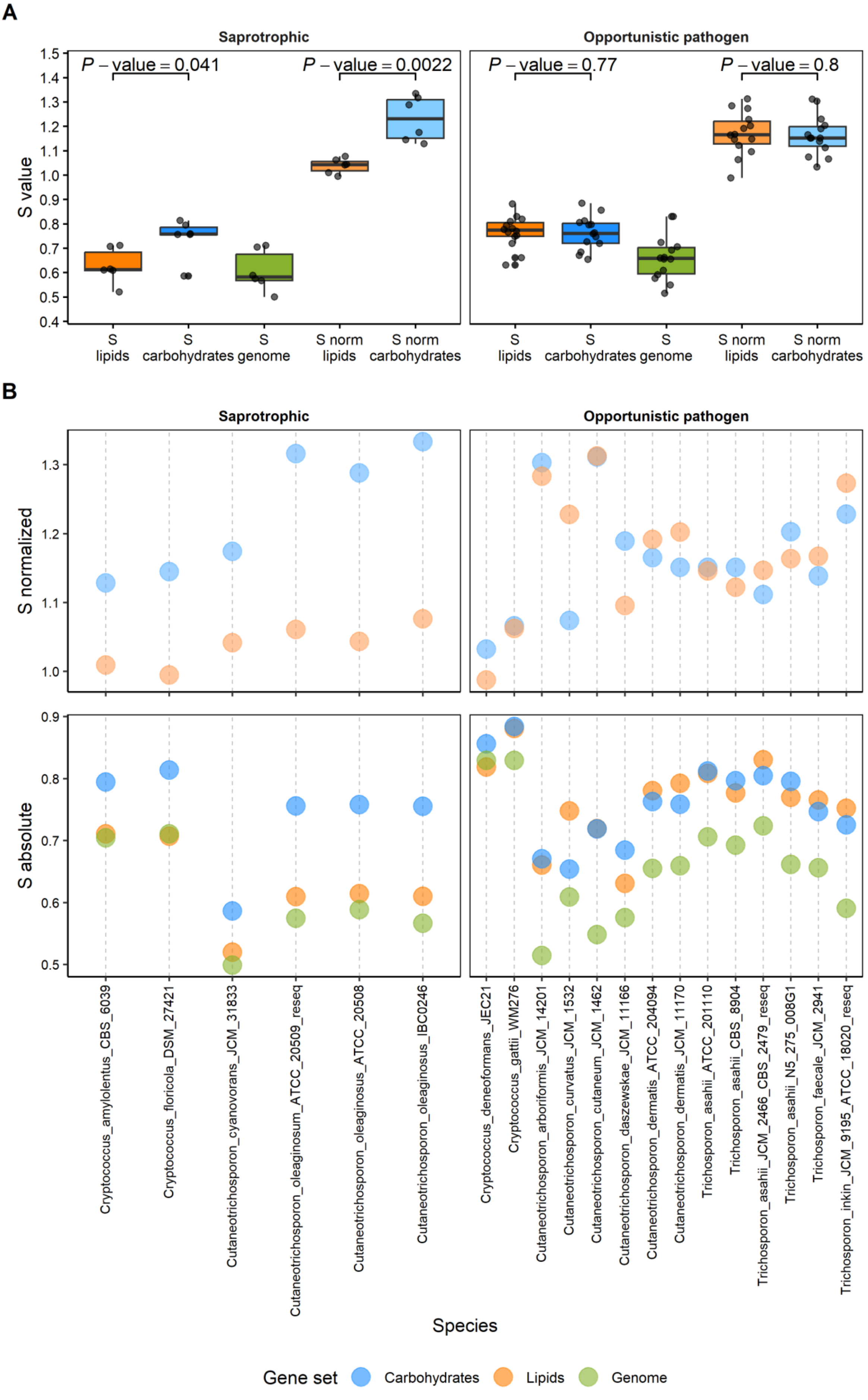
A
Saprotrophic
Opportunistic pathogen
P – value = 0.041
P – value = 0.0022
P – value = 0.77
P – value = 0.8
S value
S lipids
S carbohydrates
S genome
S norm lipids
S norm carbohydrates
B
S normalized
S absolute
Cryptococcus_amylolentus_CBS_6039
Cryptococcus_floricola_DSM_27421
Cutaneotrichosporon_cyanovorans_JCM_31833
Cutaneotrichosporon_oleaginosum_ATCC_20509_reseq
Cutaneotrichosporon_oleaginosus_ATCC_20508
Cutaneotrichosporon_oleaginosus_IBC0246
Cryptococcus_deneoformans_JEC21
Cryptococcus_gattii_WM276
Cutaneotrichosporon_arboriformis_JCM_14201
Cutaneotrichosporon_curvatus_JCM_1532
Cutaneotrichosporon_cutaneum_JCM_1462
Cutaneotrichosporon_daszewskae_JCM_11166
Cutaneotrichosporon_dermatis_ATCC_204094
Cutaneotrichosporon_dermatis_JCM_11170
Trichosporon_asahii_ATCC_201110
Trichosporon_asahii_CBS_8904
Trichosporon_asahii_JCM_2466_CBS_2479_reseq
Trichosporon_asahii_N5_275_008G1
Trichosporon_faecale_JCM_2941
Trichosporon_inkin_JCM_9195_ATCC_18020_reseq
Species
Gene set
Carbohydrates
Lipids
Genome

Supplementary Figure 17. Comparison of codon optimization (S) metrics of saprotrophic (left panel) and opportunistic pathogenic (right panel) species. The distribution of the S values is provided across gene sets (**A**) and on species level (**B**) for 3 different conditions: the absolute S value for carbohydrate or lipid transport and metabolism pathways; the overall S value for the genome; and the normalization of the S value (S norm) for each pathway in relation to the genomic S value. Each boxplot indicates the median value (center line), the 25th and 75th percentiles (lower and upper hinges), and the most extreme data points (whiskers) within 1.5 × inter-quartile range. Individual points beyond the whiskers are outliers.

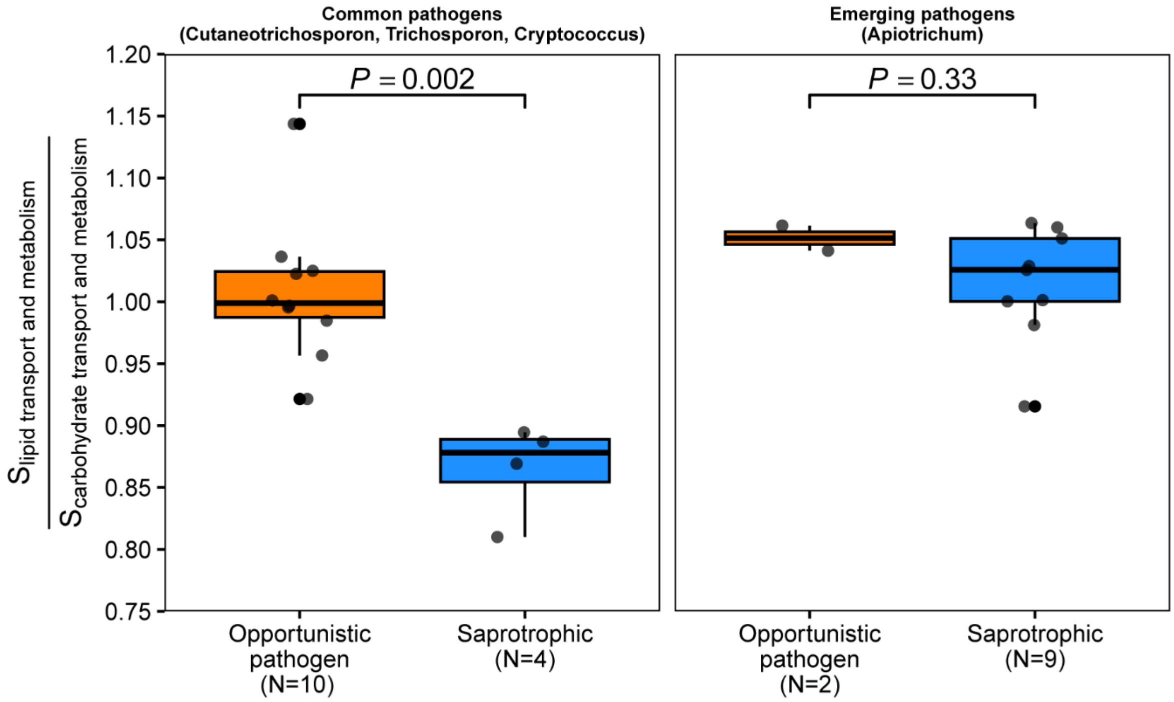

Supplementary Figure 18. Relative translation efficiency for genes involved in lipid or carbohydrate transport and metabolism with 1 isolate per species. The translation efficiency (S index) is compared between genera comprising both opportunistic pathogenic and saprotrophic species, except for *Trichosporon*. The opportunistic pathogens were grouped into common (*Cutaneotrichosporon*, *Trichosporon*, and *Cryptococcus*) or emerging (*Apiotrichum*) pathogens. These results consider only 1 genome per species and are consistent with the ones presented and are provided on Figure 3. Each boxplot indicates the median value (center line), the 25th and 75th percentiles (lower and upper hinges), and the most extreme data points (whiskers) within 1.5 × inter-quartile range. Individual points beyond the whiskers are outliers.

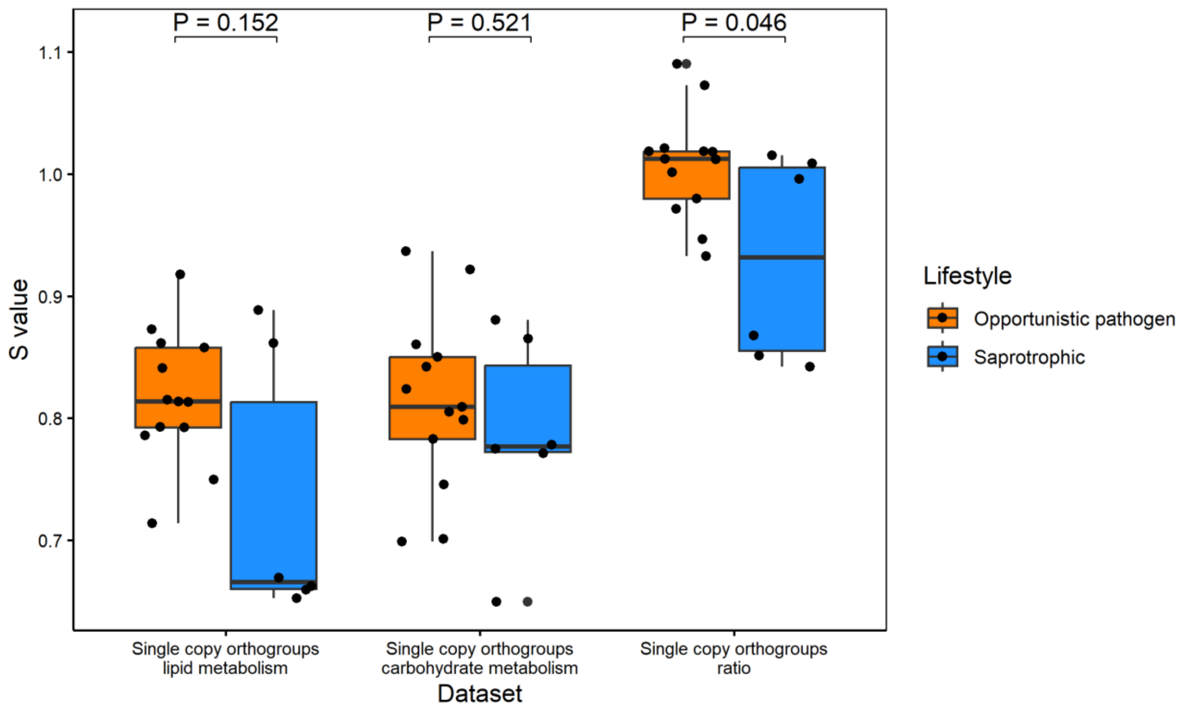

Supplementary Figure 19. Direct comparison of the codon optimization (S values) among single-copy orthogroups. Protein sequences of genes involved in carbohydrate and lipid transport and metabolism were clustered into orthogroups. The S value was determined based on single-copy orthogroups present in all tested species with well-defined lifestyles (*Cryptococcus*, *Cutaneotrichosporon*, and *Trichosporon*). The S ratio represents the relative codon optimization between both pathways (S lipid : S carbohydrate) for each species. Each boxplot indicates the median value (center line), the 25th and 75th percentiles (lower and upper hinges), and the most extreme data points (whiskers) within 1.5 × inter-quartile range. Individual points beyond the whiskers are outliers.

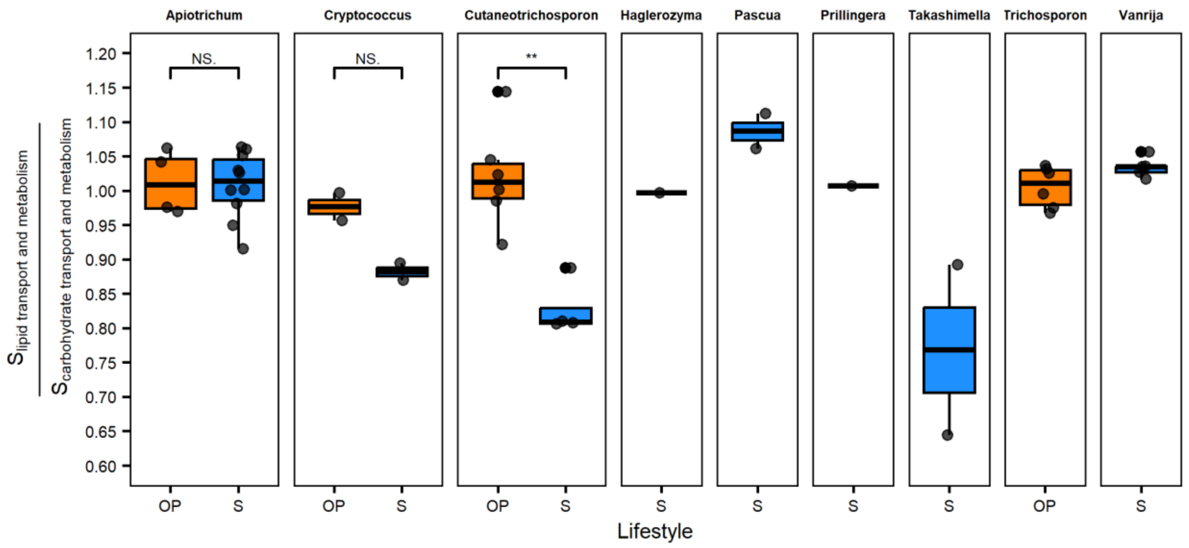

Supplementary Figure 20. Relative translation efficiency for genes involved in lipid and carbohydrate transport and metabolism. The relative translation efficiency (S index) is compared between genera and opportunistic pathogenic (OP) and saprotrophic (S) lifestyles. Each boxplot indicates the median value (center line), the 25th and 75th percentiles (lower and upper hinges), and the most extreme data points (whiskers) within 1.5 × inter-quartile range. Individual points beyond the whiskers are outliers.

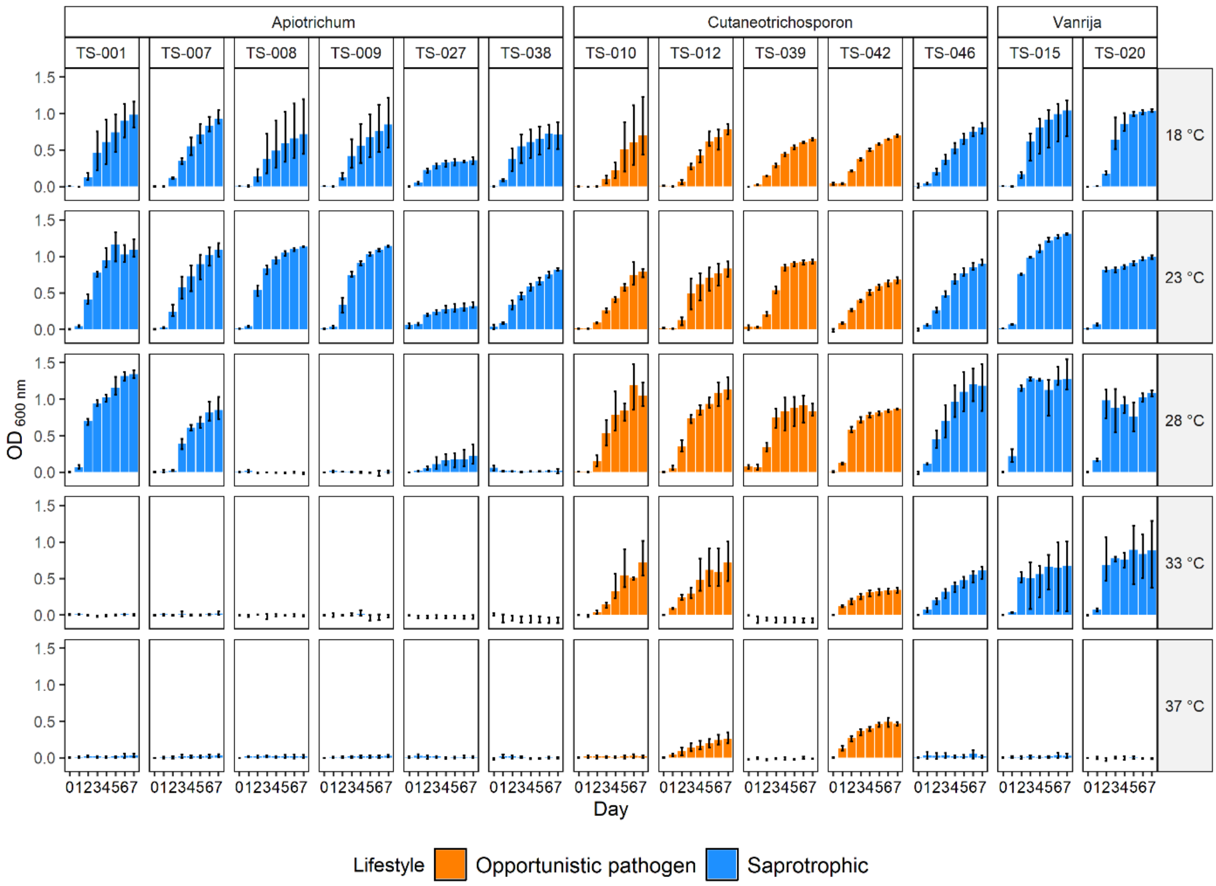

307

308 **Supplementary Figure 21**. Growth of isolates cultivated at 18°C, 23°C, 28°C, 33°C and 37°C for 10 days.

309 The optical density (OD) values at 600 nm are the mean from 4 replicates and presented excluding the

310 baseline OD of the growth medium. The isolates are separated according to genus and color-coded

311 according to lifestyle. Error bars represent the observed variation among replicates.

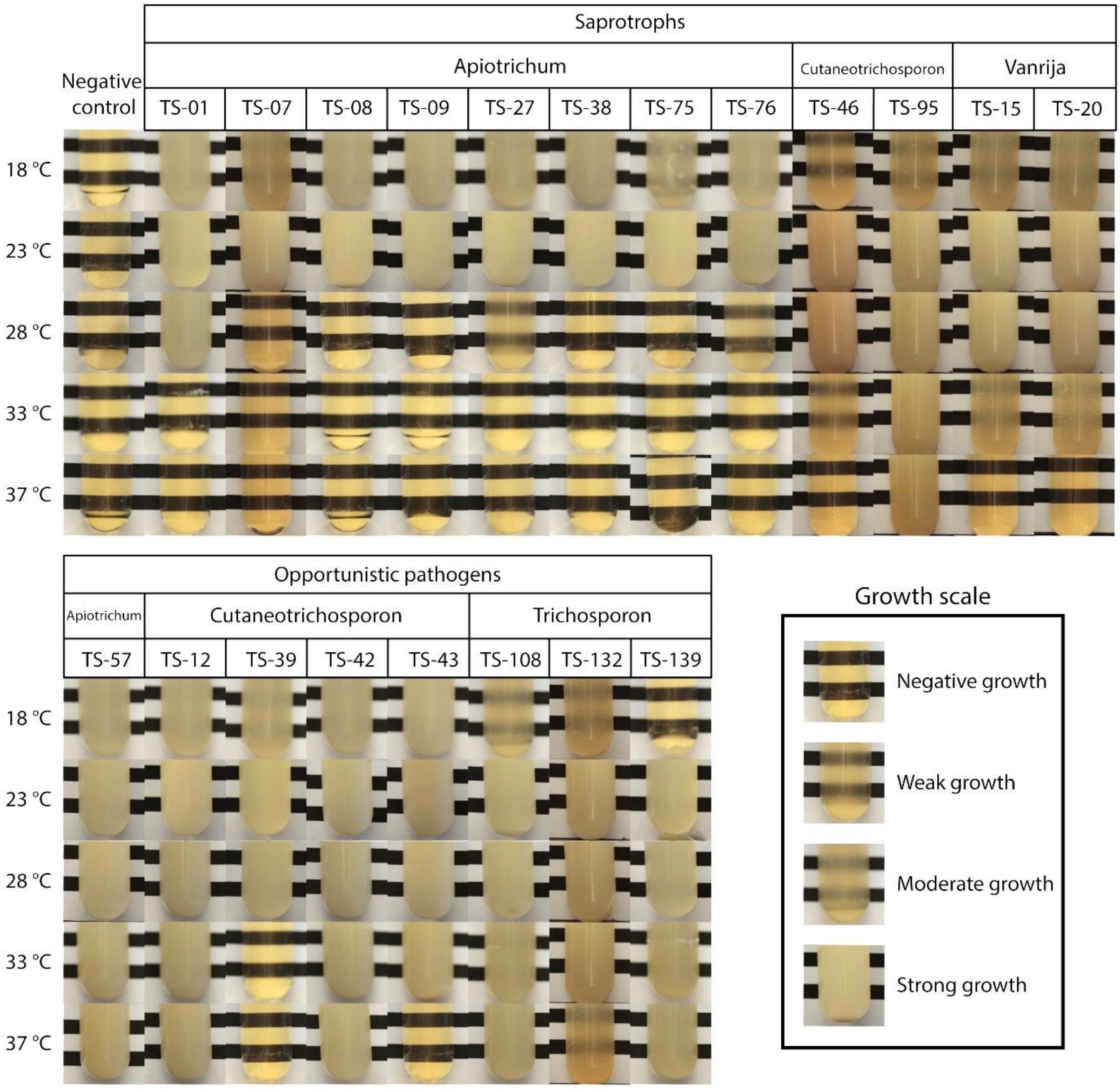

Supplementary Figure 22. Growth of isolates cultivated at 18°C, 23°C, 28°C, 33°C and 37°C for 6 days in 5 mL PD medium. Each tube corresponds to a different isolate and temperature. Qualitative growth was evaluated based on turbidity. A Wickerham card (black stripes) was placed behind each tube as a visual aid. Representative examples of growth intensity (no growth, weak, moderate and strong growth) are provided.

References

1. Marck, C. & Grosjean, H. tRNomics. Analysis of tRNA genes from 50 genomes of Eukarya, Archaea, and Bacteria reveals anticodon-sparing strategies and domain-specific features. *RNA (New York, N.Y.)* **8,** 1189–1232; 10.1017/s1355838202022021 (2002).
2. Chen, W., Xie, T., Shao, Y. & Chen, F. Genomic characteristics comparisons of 12 food-related filamentous fungi in tRNA gene set, codon usage and amino acid composition. *Gene* **497,** 116–124; 10.1016/j.gene.2012.01.016 (2012).
3. Wint, R., Salamov, A. & Grigoriev, I. V. Kingdom-Wide Analysis of Fungal Protein-Coding and tRNA Genes Reveals Conserved Patterns of Adaptive Evolution. *Molecular biology and evolution* **39**; 10.1093/molbev/msab372 (2022).
4. Wilken, S. E. *et al.* Genomic and proteomic biases inform metabolic engineering strategies for anaerobic fungi. *Metabolic engineering communications* **10,** e00107; 10.1016/j.mec.2019.e00107 (2020).
5. Cannarozzi, G. *et al.* A role for codon order in translation dynamics. *Cell* **141,** 355–367; 10.1016/j.cell.2010.02.036 (2010).
6. Huang, Y. *et al.* cncRNAdb. A manually curated resource of experimentally supported RNAs with both protein-coding and noncoding function. *Nucleic acids research* **49,** D65-D70; 10.1093/nar/gkaa791 (2021).
7. Rudinger-Thirion, J., Lescure, A., Paulus, C. & Frugier, M. Misfolded human tRNA isodecoder binds and neutralizes a 3' UTR-embedded Alu element. *Proceedings of the National Academy of Sciences of the United States of America* **108,** E794-802; 10.1073/pnas.1103698108 (2011).
8. Geslain, R. & Pan, T. tRNA. Vast reservoir of RNA molecules with unexpected regulatory function. *Proceedings of the National Academy of Sciences of the United States of America* **108,** 16489–16490; 10.1073/pnas.1113715108 (2011).
9. Goodenbour, J. M. & Pan, T. Diversity of tRNA genes in eukaryotes. *Nucleic acids research* **34,** 6137–6146; 10.1093/nar/gkl725 (2006).
10. Yona, A. H. *et al.* tRNA genes rapidly change in evolution to meet novel translational demands. *eLife* **2,** e01339; 10.7554/eLife.01339 (2013).
11. Santos, F. B. & Del-Bem, L.-E. The Evolution of tRNA Copy Number and Repertoire in Cellular Life. *Genes* **14,** 27; 10.3390/genes14010027 (2023).
12. Fijarczyk, A., Hessenauer, P., Hamelin, R. C. & Landry, C. R. Lifestyles shape genome size and gene content in fungal pathogens. *bioRxiv,* 2022.08.24.505148; 10.1101/2022.08.24.505148 (2022).
13. McDonald, M. J., Chou, C.-H., Swamy, K. B. S., Huang, H.-D. & Leu, J.-Y. The evolutionary dynamics of tRNA-gene copy number and codon-use in *E. coli*. *BMC evolutionary biology* **15,** 163; 10.1186/s12862-015-0441-y (2015).
14. Novoa, E. M., Pavon-Eternod, M., Pan, T. & Ribas de Pouplana, L. A role for tRNA modifications in genome structure and codon usage. *Cell* **149,** 202–213; 10.1016/j.cell.2012.01.050 (2012).
15. Kamilar, J. M. & Cooper, N. Phylogenetic signal in primate behaviour, ecology and life history. *Philosophical transactions of the Royal Society of London. Series B, Biological sciences* **368,** 20120341; 10.1098/rstb.2012.0341 (2013).

16. Losos, J. B. Convergence, adaptation, and constraint. *Evolution; international journal of organic evolution* **65,** 1827–1840; 10.1111/j.1558-5646.2011.01289.x (2011).

Supplementary Notes

Genomic and physiological signatures of adaptation in pathogenic fungi

Marco Alexandre Guerreiro[1,2,*], Andrey Yurkov[3], Minou Nowrousian[4], Kirk Broders[5], Eva H. Stukenbrock[1,2]

[1] Environmental Genomics Group, Botanical Institute, Christian-Albrechts University of Kiel, Kiel, Germany

[2] Max Planck Institute for Evolutionary Biology, Plön, Germany

[3] Leibniz Institute DSMZ-German Collection of Microorganisms and Cell Cultures, Braunschweig, Germany

[4] Department of Molecular and Cellular Botany, Ruhr University Bochum, Bochum, Germany

[5] USDA, Agricultural Research Service, National Center for Agricultural Utilization Research, Mycotoxin Prevention and Applied Microbiology Research Unit, 1815 N. University, Peoria, IL. 61604, U.S.A

*** Corresponding author**: Marco Alexandre Guerreiro, Max Planck Institute for Evolutionary Biology, August-Thienemann-Str. 2, 24306 Plön and Christian-Albrechts University of Kiel, Am Botanischen Garten 1-9, 24118 Kiel

Phone: +49 (0) 431 880 6366, Fax: +49 (0) 431 880 6369

Email: mguerreiro@bot.uni-kiel.de

Supplementary Note 1

tRNA composition and expansion among *Trichosporonales* are independent from the phylogenetic structure

Our comparative analyses revealed that the total number of tRNA genes (tRNAome) varied largely across the 41 analyzed *Trichosporonales* genomes (Fig. 1 and Supplementary Data 6). The mean tRNAome comprised 444 genes, with a minimum of 51 and a maximum of 1455 tRNA genes (Supplementary Fig. 5). *Apiotrichum laibachii* and *Ap. akiyoshidainum* possessed unusually large tRNAomes with 1455 and 1227 genes, respectively, while *Takashimella koratensis* and *Ta. tepidaria* possessed comparatively much smaller tRNAomes with 51 and 58 genes, respectively (Supplementary Data 6). These analyses revealed an uneven, wide distribution of tRNA gene content among the phylogeny.

We next sought to understand if the distinct tRNA composition has functional relevance. We correlated tRNA composition with codon usage throughout the fungal genomes. First, we investigated the composition of anticodon types and found a considerable number of unique anticodon types (i.e., tRNA gene families), ranging from 39 in *Ap. porosum* (JCM 1458) and *Ta. koratensis* to 46 in *Ap. mycotoxinovorans* and *Haglerozyma chiarellii* (from the universal 61 possible anticodons) (Supplementary Fig. 6 and Supplementary Data 6). Nevertheless, we detected tRNAs decoding all 20 universal amino acids in most genomes (Supplementary Fig. 7 and Supplementary Data 6). However, tRNAs genes decoding histidine ($tRNA^{His}$) were in low abundance overall and even absent in 15 genomes (*Ap. domesticum*, *Ap. montevideense*, *Ap. porosum*, *Cutaneotrichosporon arboriformis*, *Cu. curvatus*, *Cu. cyanovorans*, *Cu. dermatis*, *P. fragicola*, *Trichosporon asahii*, and *Tr. inkin*), while the aspartate-encoding tRNA ($tRNA^{Asp}$) was absent in *Pascua* and *Takashimella* spp. Some of these genes were, however, detected as pseudogenes during the tRNA prediction (Supplementary Data 7). Additionally, certain low copy number genes were exclusive to some species (e.g., Ala-GGC present only in *Ap. gamsii*) or clades (e.g., Ser-ACU was only detected in 1 sub-clade of *Cutaneotrichosporon*

species). Interestingly, we observed that only 1 tRNA gene family per amino acid was expanded across different species (Supplementary Fig. 7 and Supplementary Data 6). Surprisingly, the most frequent tRNA gene families did not consistently correspond with the most frequently utilized synonymous codon in the overall coding sequences (Supplementary Data 8). Intriguingly, the most common synonymous codons often corresponded to tRNA gene families absent in the genome.

To understand if the evolutionary process of tRNA expansion correlates with the phylogenetic relationship of species, we used Blomberg's K measure to assess evolution of the trait (in this case, gene copy number) considering phylogenetic distance as the only predictor of trait similarity among species. Additionally, we tested the pairwise similarity and correlation of gene composition among species considering their phylogenetic distance.

A tRNA gene composition (i.e., copy number per gene family) of 45 out of 55 tRNA genes produced a statistically significant phylogenetic signal (Blomberg's P <0.05). Most genes (38 out of 45) exhibited a low phylogenetic signal (Blomberg's K <1, Blomberg's P <0.05) (Supplementary Fig. 8), suggesting a reduced phylogenetic signal in the distribution of genes, potentially due to convergent evolution, because closely related species are less similar than expected in comparison to distantly related species under a Brownian motion model of trait evolution. In contrast, only five genes showed a high phylogenetic signal (Blomberg's K >1, Blomberg's P <0.05), indicating stabilizing selection or low rates of evolution related to gene copy number, suggesting that this trait is highly conserved phylogenetically and that closely related species are more similar than expected based on phylogenetic relationships. The lack of a significant phylogenetic signal (Blomberg's P >0.05) indicates that the remaining 2 genes have reduced occurrence in the phylogeny or that they evolved independently of the phylogenetic structure. Furthermore, tRNA gene composition similar among distantly related species (Supplementary Fig. 9), however, our analyses revealed tRNA gene expansion, copy number, and composition to be dependent from the phylogenetic structure.

Additionally, our results suggest a dynamic tRNA repertoire in which tRNA gene expansion occurred multiple times in this group of species, which cannot be solely explained by lifestyle.

Supplementary Note 2

Intragenomic tRNA gene sequence variation suggests recent and multiple gene duplication events

To gain a better understanding of the observed tRNA expansion (Fig. 1, Supplementary Data 6 and Supplementary Fig. 7), we further assessed the intragenomic sequence variation of multiple-copy tRNA genes. First, we investigated the relevance of gene copy number to variation within genomes for each gene family (i.e., genes with the same anticodon sequence). Here, we expected to find 2 possible scenarios: (1) genes with high copy number would show a high level of similarity, suggesting recent gene duplication events, while genes with low copy number would be more variable, suggesting ancient duplications (supporting Supplementary Fig. 8 and Supplementary Fig. 9); or (2) high similarity of low copy number genes due to high functional selective pressure, with relaxed selection on high copy number genes due to functional gene redundancy.

Among *Trichosporonales*, the nucleotide variation (transitional and transversional substitution rates based on Kimura's 2-parameter distance) in each tRNA gene family was inversely proportional to gene copy number (Supplementary Fig. 11A). tRNA genes with a high copy number showed low sequence variation, while genes with low copy number displayed the highest intragenomic variation (Supplementary Fig. 11B). Variable positions among gene copies were mainly located in the predicted intronic region, but nucleotide substitutions and insertion/deletions were also detected in the tRNA body. These results support the hypothesis of a recent gene expansion for the highly abundant tRNA gene families.

Next, we compared nucleotide variation among all tRNA gene families within each genome to assess whether some tRNAs exhibit more variation than others. Redundant tRNA genes decoding the same amino acid display different levels of variation. When amino acids were decoded by multiple genes, one of the genes displayed a higher level of conservation than the others (e.g., $tRNA^{Arg}$-ACG and $tRNA^{Arg}$-UCU) (Supplementary Fig. 12). The tRNA with the highest intragenomic sequence variation

was for the tRNA^{Leu}-UAG gene (median distance: 0.76), while tRNA^{Gly}-GCC was the most conserved gene (median distance: 0.02) (Supplementary Fig. 12 and Supplementary Fig. 13). These results suggest a differential selective pressure among genes decoding the same amino acid and also across genes decoding different amino acids.

Supplementary Note 3

Interspecific tRNA gene sequence variation reveals different selective patterns

To address the selective pressure acting on tRNA genes across the phylogeny, we compared the overall tRNA nucleotide variation among all members of *Trichosporonales*. Different species carried different levels of nucleotide sequence variation in their tRNA genes (Supplementary Fig. 14). The highest rate of tRNA sequence variation was detected in *Takashimella koratensis* (median distance of 0.32 per tRNA gene), while *Cutaneotrichosporon curvatus* exhibited the least variation (median distance of 0.06 per tRNA gene). No statistical significance was detected between intraspecific tRNA gene copy variation and lifestyles (Wilcoxon test $P \geq 0.05$). These results suggest different selective pressure for each species independent of the phylogenetic structure and lifestyle. Furthermore, this could indicate that multiple independent gene duplication events occurred throughout the evolutionary history of this order, some being very recent.
