# Supplementary material for: Genomic and physiological signatures of adaptation in pathogenic fungi
Source: Nat Commun. 2026 Jan 15;17:748. doi: 10.1038/s41467-026-68330-6 (PMC12820176; doi:10.1038/s41467-026-68330-6)
Supplement: Supplementary file 4 — Description of Additional Supplementary Files [file 41467_2026_68330_MOESM4_ESM.pdf]

Description of Additional Supplementary Files

File Name: Supplementary Data 1

Description: Isolates and genome assemblies analyzed in this study and respective relevant metadata are provided. The status of the strain is indicated for type strains (T) and hybrids (H). Accession numbers from NCBI and links for JGI and ATCC for the genome assemblies are provided. When available, accession numbers for additional genome assemblies for the same strain are provided in parentheses. The SRA experiments are indicated for the genomes for which the molecular barcodes were retrieved from the raw reads data. Strain IDs used throughout the text and figures are highlighted in bold, while individuals selected as species representatives for statistical analyses are underlined.

File Name: Supplementary Data 2

Description: PERMANOVA (two-sided) results for statistical tests using different datasets. The subset of genes or genomic metrics is indicated as the dataset, along with the corresponding PERMANOVA formula. The phylogenetic structure was decomposed into principal components (PC) and tested alongside lifestyle. The degrees of freedom (Df), coefficient of determination (R2), and F-value are provided. Significant p-values are highlighted in bold.

File Name: Supplementary Data 3

Description: EggNOG annotation for genes involved in carbohydrate and lipid transport and metabolism.

File Name: Supplementary Data 4

Description: Function and distribution of orthologous genes related to lipid transport and metabolism exclusively detected for each lifestyle.

File Name: Supplementary Data 5

Description: Function and distribution of orthologous genes related to carbohydrate transport and metabolism exclusively detected for each lifestyle.

File Name: Supplementary Data 6

Description: Distribution of tRNA genes among Trichosporonales fungi on the genome assembly data. The overall number of detected genes is shown by anticodon and the corresponding decoded number of amino acids. The total number of genes per decoded amino acid and anticodon is also provided.

File Name: Supplementary Data 7

Description: Distribution of tRNA genes classified as pseudogenes among Trichosporonales fungi on the genome assembly data. The overall number of detected pseudogenes is shown

by anticodon and the corresponding decoded number of amino acids. The total number of genes per decoded amino acid and anticodon is also provided. All the tRNA classified pseudogenes were excluded from further analyses.

File Name: Supplementary Data 8

Description: Codon usage and anticodon frequency in Trichosporonales and Tremellales. The relative synonymous codon usage (RSCU) is provided for the genome and for carbohydrate and lipid metabolic pathways. The frequency of the respective decoding tRNA gene family is provided. The most common codon and most frequent tRNA gene family are bolded for each amino acid.

File Name: Supplementary Data 9

Description: S values and respective p-value for all the considered pathways. Pvalues are based on Pearson correlations as determined by the tAI R package.

File Name: Supplementary Data 10

Description: S and p-values for single-copy orthologous genes (OG) present in all species predicted to be involved in Carbohydrate Transport and Metabolism (carbohydrates) or Lipid Transport and Metabolism (lipid). P-values are based on Pearson correlations as determined by the tAI R package.

File Name: Supplementary Data 11

Description: Accuracy of fungal lifestyle prediction based on S ratio, for each isolate, based on 9999 iterations of the decision tree model trained on a partial dataset.

File Name: Supplementary Data 12

Description: Isolates used in this study for the growth assays.

File Name: Supplementary Data 13

Description: Colony Forming Units (CFU) counts per isolate in triplicate at an OD_{600nm} of 2.5×10^{-4}.

File Name: Supplementary Data 14

Description: Functional annotation for all genes predicted. Functional prediction was performed with EggNOG. The predicted tAI is provided for each gene. The isolates used as representatives for individual species are indicated.
